# LINC01468 drives NAFLD-HCC progression through CUL4A-linked degradation of SHIP2

**DOI:** 10.1038/s41420-022-01234-8

**Published:** 2022-11-07

**Authors:** Hongquan Wang, Yan Wang, Shihui Lai, Liang Zhao, Wenhui Liu, Shiqian Liu, Haiqiang Chen, Jinhua Wang, Guanhua Du, Bo Tang

**Affiliations:** 1 Department of Pancreatic Cancer, Tianjin Medical University Cancer Institute and Hospital, National Clinical Research Center for Cancer, Key Laboratory of Cancer Prevention and Therapy, Tianjin’s Clinical Research Center for Cancer, 300060 Tianjin, P. R. China; 2 Department of Hepatobiliary Surgery, The First Affiliated Hospital of Guangxi Medical University, 530021 Nanning, Guangxi People’s Republic of China; 3 Beijing Key Laboratory of Drug Target and Screening Research, Institute of Materia Medica, Chinese Academy of Medical Sciences and Peking Union Medical College, 100050 Beijing, P. R. China

**Keywords:** Experimental models of disease, Hepatocellular carcinoma

## Abstract

Accumulating evidence suggests that long noncoding RNAs (lncRNAs) are deregulated in hepatocellular carcinoma (HCC) and play a role in the pathogenesis of non-alcoholic fatty liver disease (NAFLD). However, the current understanding of the role of lncRNAs in NAFLD-associated HCC is limited. In this study, transcriptomic profiling analysis of three paired human liver samples from patients with NAFLD-driven HCC and adjacent samples showed that LINC01468 expression was significantly upregulated. In vitro and in vivo gain- and loss-of-function experiments showed that LINC01468 promotes the proliferation of HCC cells through lipogenesis. Mechanistically, LINC01468 binds SHIP2 and promotes cullin 4 A (CUL4A)-linked ubiquitin degradation, thereby activating the PI3K/AKT/mTOR signaling pathway, resulting in the promotion of de novo lipid biosynthesis and HCC progression. Importantly, the SHIP2 inhibitor reversed the sorafenib resistance induced by LINC01468 overexpression. Moreover, ALKBH5-mediated N^6^-methyladenosine (m^6^A) modification led to stabilization and upregulation of LINC01468 RNA. Taken together, the findings indicated a novel mechanism by which LINC01468-mediated lipogenesis promotes HCC progression through CUL4A-linked degradation of SHIP2. LINC01468 acts as a driver of HCC progression from NAFLD, highlights the potential of the LINC01468-SHIP2 axis as a therapeutic target for HCC.

## Introduction

Hepatocellular carcinoma (HCC), the most common primary liver cancer, is considered the second-most common cause of cancer-related death globally and is the fifth-most common cancer worldwide [1]. HCC is known to be caused by cirrhosis resulting from chronic infection (hepatitis B virus and hepatitis C virus) and alcohol-induced injury [2]. However, despite the reduction in the incidence of chronic infection-related HCC with the development of anti-HCV drugs and effective vaccines for HBV [1], HCC-associated mortality has been rising prominently, suggesting that other risk factors likely account for this increase.

With a global rise in type 2 diabetes (T2DM) and obesity, non-alcoholic fatty liver disease (NAFLD), now known as metabolic dysfunction-associated fatty liver disease (MAFLD) [3, 4], is becoming an increasingly important etiology of HCC [5, 6]. NAFLD is considered to indicate a metabolic predisposition to liver cancer [7], and is now becoming the dominant cause of HCC worldwide [8]. However, the molecular mechanisms underlying the progression of NAFLD to HCC remain largely unknown [9, 10].

Long noncoding RNAs (lncRNAs) are a novel class of RNAs >200 nucleotides in length that lack the ability to encode proteins. lncRNAs are deregulated in HCC and exert crucial roles in the occurrence and progression of HCC [11], and some lncRNAs act as vital metabolic regulators that are involved in the etiology of NAFLD [12–15]. Although lncRNAs may contribute to the progression of NAFLD and HCC [16–20], their role in NAFLD-associated HCC is not well-understood, indicating the need to delineate the relevant mechanisms underlying NAFLD-HCC progression.

LINC01468 is a newly identified lncRNA [21, 22] that functions as an oncogene contributing to the progression of lung adenocarcinoma [23]. However, the roles and underlying mechanisms of LINC01468 in HCC remain unclear, and the role of LINC01468 in NAFLD-related HCC has not yet been reported. In this study, we identified significant upregulation of LINC01468 in NAFLD and HCC. LINC01468 silencing inhibited HCC tumorigenesis via lipid metabolism and suppressed the chemoresistance of HCC cells. Mechanistically, LINC01468 directly interacted with SHIP2 and destabilized SHIP2 by enhancing E3 ubiquitin ligase cullin 4 A (CUL4A) ubiquitination-dependent SHIP2 degradation. Taken together, the findings of the present study revealed a new mechanism by which LINC01468-mediated lipogenesis promotes hepatocellular carcinoma progression through the CUL4A-linked degradation of SHIP2.

## Results

### LINC01468 is especially upregulated in NAFLD-associated HCC

**Fig. 1:**
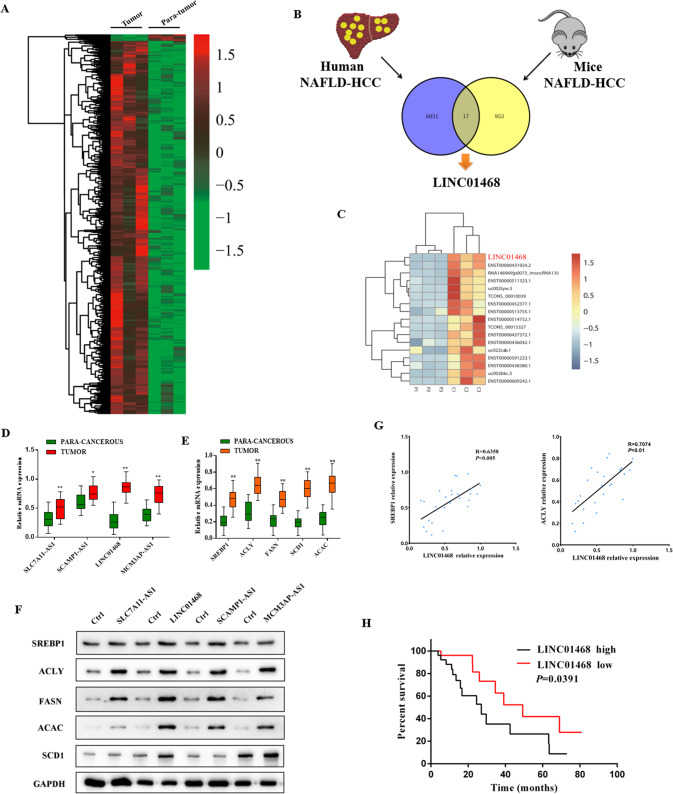
LINC01468 is especially upregulated in liver tissues during NAFLD-HCC. **A** Heatmap summarizing RNA-Seq data for human HCC with or without NAFLD with 5944 upregulated genes (pink) and 104 downregulated genes (green). **B** Overlapping of the 17 genes significantly altered in both human (*n* = 3) and mice (*n* = 3) NAFLD-HCC (*P* < 1.78 × 10^−9^). **C** Part of the genes described in **B**. **D** qRT-PCR was used to assess the relative expression levels of four lncRNAs in 26 paired peritumoral tissues and HCC tissues (**P < 0.01)^.^ **E** qRT-PCR was used to determine the levels of lipogenic enzymes and prolipogenic transcription factor in 26 paired HCC tissues and peritumoral tissues. **F** Representative immunoblotting results for the major lipogenesis protein with or without the indicated LncRNA overexpression in Huh7 cell line. **G** Scatter-plot analysis of the correlation between the mRNA levels of LINC01468 and SREBP1 or ACLY in 26 HCC tissues. **H** Kaplan–Meier analysis of the correlation of LINC01468 expression with overall survival.

To reveal the role of lncRNA in NAFLD-associated HCC, we first analyzed three paired human liver tumor tissues and adjacent normal tissues (*n* = 3) from patients with NAFLD-driven HCC by RNA-seq. In comparison with paired adjacent normal tissues, 5944 genes were upregulated and 104 were downregulated in NAFLD-HCC (Fig. 1A and Supplementary Fig. 1A). An analysis of the differentially expressed genes in human NAFLD-HCC showed a significant overlap of 17 genes with those in mice NAFLD-HCC (Fig. 1B). The results of hierarchical clustering and heatmap analysis of the significantly differentially expressed genes between human and mouse NAFLD-HCC are shown in Fig. 1C. Overexpression of lncRNAs in HCC tissues was confirmed by qRT-PCR with 26 paired samples. LINC01468 was the most significantly upregulated among the four lncRNAs, and showed the highest log2 fold-change values (Fig. 1D). The expression of lipogenic pathway enzymes such as SREBP1, FASN, ACLY, ACAC, and SCD1 was detected by qRT-PCR, and progressive induction of SREBP1, ACLY, FASN, ACAC, and SCD1 was observed in HCC tissues (Fig. 1E). To explore the functions of the four selected lncRNAs in HCC, we investigated their effects on the expression of lipogenic pathway enzymes. Overexpression of lncRNAs upregulated the protein-level expression of lipogenic pathway enzymes in HCC cell lines, with LINC01468 inducing a significant upregulation (Fig. 1F). Scatter-plot analysis indicated a positive correlation between the mRNA levels of LINC01468 and SREBP1 (r = 0.6358, *p* < 0.005), ACLY (r = 0.7074, *p* < 0.01) (Fig. 1G), SLC7A11-AS1, SREBP1 (r = 0.5280, *p* = 0.0056), and ACLY (r = 0.4271, *p* = 0.0296) (Supplementary Fig. 1B), SCAMP1-AS1, SREBP1 (r = 0.4783, *p* = 0.0134), and ACLY (r = 0.4202, *p* = 0.0326) (Supplementary Fig. 1C), MCM3AP-AS1 and SREBP1 (r = 0.4963, *p* < 0.001), and ACLY (r = 0.4724, *p* = 0.0148) (Supplementary Fig. 1D). Next, the correlation between LINC01468 expression and clinicopathological findings in 52 NAFLD-HCC cases was examined. Based on the median expression levels of LINC01468 detected by qRT-PCR, patients were divided into two groups. LINC01468 levels were significantly related to hemoglobin A1C (HbA1C), triglyceride (TG), and total cholesterol (TC), and cirrhosis levels, tumor size, tumor stage, TNM stage, and microvascular invasion (Table 1). We further examined the correlation between LINC01468 expression and the 5-year follow-up data of the patients. Patients with high LINC01468 expression showed a significantly lower overall survival when the median LINC01468 expression level in 52 patients was used as the cutoff point (Fig. 1H).

**Table 1:** Relationship between LINC01468 and clinicopathological parameters in 52 HCC patients.

Variables	All cases	LINC01468 expression		*P*
		Low (*n* = 26)	High (*n* = 26)	
Age (years)				0.768
<50	35	17	18	
≥50	17	9	8	
Gender				0.150
Male	33	14	19	
Female	19	12	7	
HbA1C				0.032
<6.5%	37	22	15	
≥6.5	15	4	11	
Cirrhosis				0.011
No	21	15	6	
Yes	31	11	20	
Tumor size (cm)				0.048
<5	21	14	7	
≥5	31	12	19	
TNM stage				0.026
I–II	28	18	10	
III–IV	24	8	16	
Microvascular invasion				0.012
Yes	25	8	17	
No	27	18	9	
AFP, μg/L				0.578
<200	24	13	11	
≥200	28	13	15	
TG				0.048
Yes	40	23	17	
No	12	3	9	
TC				0.039
Yes	35	21	14	
No	17	5	12	

### Silencing LINC01468 inhibits HCC chemoresistance and tumorigenesis

**Fig. 2:**
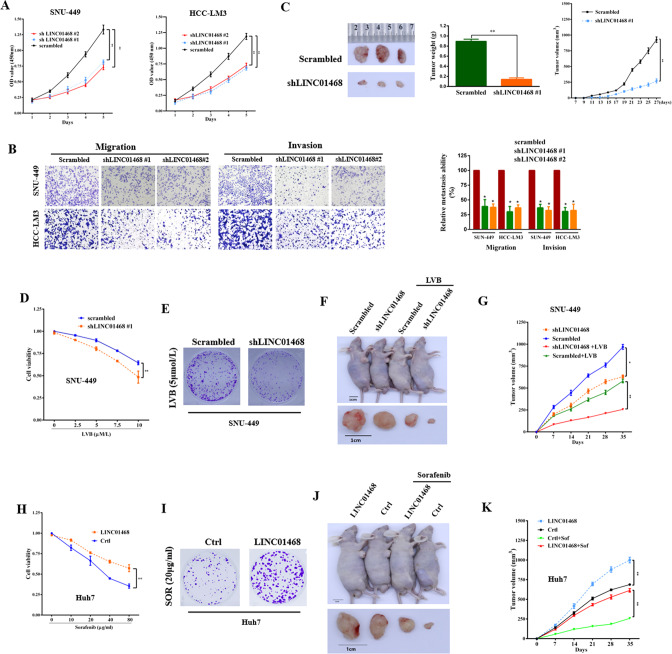
LINC01468 silencing suppresses the chemoresistance of HCC cells and inhibits HCC tumorigenesis by lipid metabolism. **A** CCK8 assays in SNU-449 and HCC-LM3 cells transfected with or without shLINC01468. **B** Representative images (left panel) and number (right panel) of migratory or invasive cells transfected with the scrambled control or shLINC01468. **C** Representative images (left panel), weight (middle panel), and growth (right panel) of xenografts derived from HCC cells stably transfected with the scrambled control or shLINC01468. **D** The viability of SNU-449 cells transfected with shLINC01468 or scrambled control in the presence of different concentrations of lenvatinib (LVB) treatment was examined by the CCK8 assay. **E** A representative image of colony formation of SNU-449 cells transfected with shLINC01468 or scrambled control after treatment with 5 μM LVB. **F**, **G** Representative tumor images (**F**) and tumor growth curves (**G**) of xenografts derived from SNU-449 cells stably transfected with shLINC01468 or scrambled control in the presence or absence of intraperitoneal LVB injections. The right flanks of all the mice were subcutaneously injected with 5 × 10^6^ cells. The tumors were collected after 4 weeks. **H** Viability of Huh7 cells transfected with LINC01468 or Ctrl in the presence of different concentrations of sorafenib was examined by the CCK8 assay. **I** A representative image of colony formation of Huh7 cells transfected with LINC01468 or Ctrl after treatment with 20 μg/mL of sorafenib. **J**, **K** Representative tumor images (**J**) and tumor growth curves (**K**) of xenografts derived from Huh7 cells stably transfected with LINC01468 or control in the presence or absence of intraperitoneal injections of 20 μg/mL sorafenib. The right flanks of the mice were subcutaneously injected with 5 × 10^6^ cells. The tumors were collected after 4 weeks. The data represent the means ± S. D (*P < 0.05; **P < 0.01).

Considering the upregulation of LINC01468 expression in NAFLD-HCC, we explored the function of LINC01468 in HCC. LINC01468 silencing reduced the proliferative capacity of HCC cells (Fig. 2A, all *P* < 0.01), and LINC01468 knockdown inhibited the migration and invasion of HCC cells (Fig. 2B, all *P* < 0.05). Similarly, LINC01468 knockdown reduced the tumorigenesis of HCC cells in vivo, indicating that HCC cells with LINC01468 knockdown showed slower and less sustainable tumor growth in the xenograft model than in the scrambled control group (Fig. 2C). Overall, these findings indicate that LINC01468 promotes HCC development through lipid accumulation.

Reprogramming of lipid metabolism is closely related to drug resistance in cancer [24]. Therefore, we assessed the effects of LINC01468 on lenvatinib (LVB) and sorafenib (SOR) sensitivity. Sorafenib was the first multi-tyrosine kinase inhibitor approved for the treatment of patients with unresectable HCC [25], while lenvatinib is another tyrosine kinase inhibitor that received approval for first-line treatment of patients with advanced HCC [26]. LINC01468 silencing sensitized SNU-449 cells to LVB, as reflected by a reduction in cell viability (Fig. 2D), colony formation (Fig. 2E), and tumorigenicity (Fig. 2F, G). Concurrently, exogenously overexpressing LINC01468 reduced the sensitivity of Huh7 cells to SOR (Fig. 2H–K). Together, these results suggest that LINC01468 promotes HCC proliferation and metastasis, thereby conferring drug chemoresistance.

### LINC01468 activates Akt/mTOR signaling pathway

**Fig. 3:**
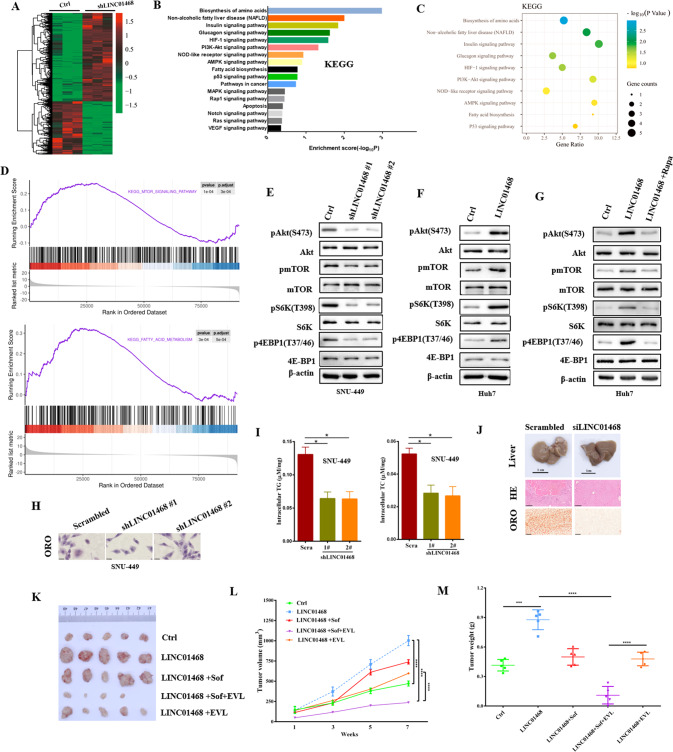
LINC01468 activates Akt/mTOR signaling pathway. **A** Differently expressed genes in SNU-449 cells transfected with shLINC01468 or control were shown by heatmap. **B** KEGG pathway analysis of LINC01468-regulated genes. **C** KEGG pathway enrichment analysis of target genes by a bubble chart. The size and color of the dots represents the number and level of genes that were enriched in the corresponding pathways, respectively. **D** The item was enriched using GSEA in SUN-449 cells transfected with shLINC01468 or control. **E**, **F** Immunoblot detection of Akt/mTOR signaling pathway protein level in **E** LINC01468-knockdown SNU-449 cells or **F** LINC01468-overexpressed Huh7 cells. **G** Immunoblotting of the indicated protein lysates from Huh7 cells expressing Ctrl or LINC01468 treated with or without rapamycin (Rapa). **H** The intracellular lipid droplets in SUN-449 cells transfected with shLINC01468 or control were stained with oil red O. **I** The cellular content of triglycerides and phospholipids in the indicated cells in **H** was detected. **J** Representative liver images (top), H&E staining (middle), and ORO staining (bottom) of xenografts derived from SUN-449 cells stably transfected with the scrambled control or siLINC01468. **K**–**M** Overexpression of LINC01468 increased tumor growth and mediated sorafenib resistance of HCC tumors in nude mice. Images show representative tumor (**K**). Growth curves (**L**) and tumor weights (**M**) of mean ± SD of six mice in each group.***P < 0.005; ****P < 0.001.

To explore the functions of LINC01468 in HCC, we performed RNA-seq in HCC cells transfected with shLINC01468 or a scrambled control. A total of 2056 unique transcripts were identified using three independent biological replicates, including 1345 upregulated and 711 downregulated mRNAs (Fig. 3A). KEGG pathway enrichment analysis suggested that these genes, including NAFLD genes, were enriched in cancer-related pathways (Fig. 3B and C). The differentially expressed gene sets were related to mammalian target of rapamycin (mTOR) and fatty acid (FA) metabolism, which showed a significantly positive correlation with LINC01468 expression in the gene set enrichment analysis (GSEA), indicating a pivotal role of LINC01468 in lipid metabolism regulation (Fig. 3D). To confirm that LINC01468 regulates mTOR, we investigated the effect of LINC01468 disruption on the expression of the Akt/mTOR pathway. LINC01468 knockdown decreased protein expression of the Akt/mTOR pathway (Fig. 3E), and the protein levels of the Akt/mTOR pathway increased after LINC01468 overexpression (Fig. 3F). Moreover, the mTORC1 inhibitor rapamycin significantly diminished the activation of the Akt/mTOR pathway by LINC01468 overexpression (Fig. 3G). We also evaluated the effect of LINC01468 on the lipid content in HCC. As shown in Fig. 3H, LINC01468 silencing significantly decreased the level of neutral lipid staining by oil red O. These results were further corroborated by the findings for the cellular lipid content, indicating that LINC01468 silencing significantly decreased the levels of intracellular TG and TC (Fig. 3I). LINC01468 silencing had significantly decreased levels of neutral lipid in vivo (Fig. 3J). To examine the functional consequences of LINC01468 in vivo, we established orthotopic xenografts derived from control- or LINC01468-expressing HCCs. Tumors overexpressing LINC01468 grew faster than those in the control group and became resistant to sorafenib (Fig. 3K). The mTOR pathway is involved in many hallmarks of cancer, including cell growth, metabolic reprogramming, proliferation, and inhibition of apoptosis, and is upregulated in HCC tissue samples. Pharmacological inhibition of the mTOR pathway (e.g., by rapamycin or everolimus) can hamper tumor progression both in vitro and in animal models. Everolimus, an mTOR inhibitor, exhibits antitumor activity by disrupting various signaling pathways [27], and has been studied in combination with sorafenib in patients with unresectable or metastatic HCC [28]. Sorafenib combined with everolimus (an mTOR inhibitor) significantly reduced tumor growth and restored sensitivity to sorafenib therapy in LINC01468-overexpressing tumors (Fig. 3K, L, and M).

### LINC01468 directly interacts with SHIP2

**Fig. 4:**
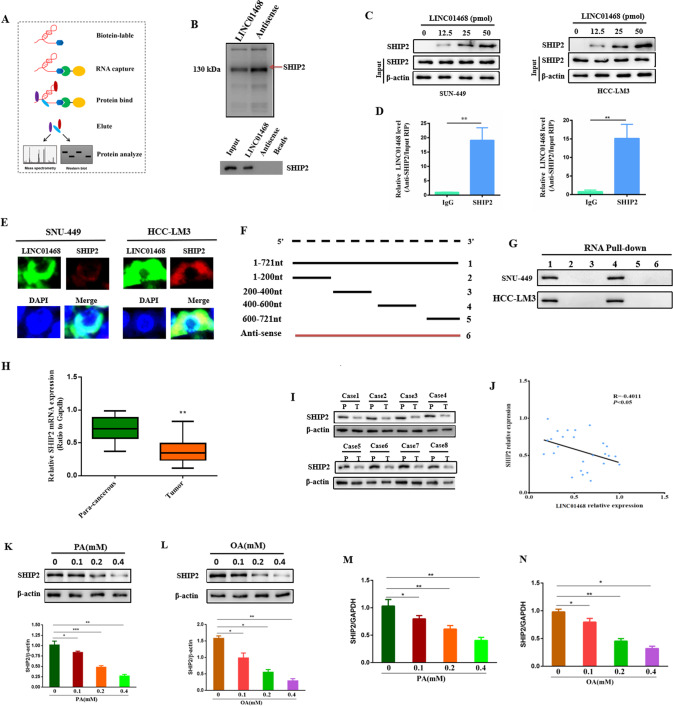
LINC01468 directly binds with SHIP2. **A** RNA pull-down assay flowchart was used to identify LINC01468-associated proteins. **B** The biotinylated LINC01468-associated proteins were stained with silver, and the bands specifically precipitated by LINC01468 were excised and submitted for mass spectrometry (top) and western blot (bottom) analysis. The arrows indicate SHIP2 proteins as the unique bands for LINC01468. **C** The interaction of SHIP2 and LINC01468 was analyzed by western blot. **D** RIP assay was performed to detect LINC01468 enrichment in the immunoprecipitated complexes using anti-SHIP2 antibodies. **E** FISH and IF assays were performed to determine the co-localization of LINC01468 (Cy3; red) and SHIP2 (green) in cells. Nuclei, blue (DAPI). **F** Diagrams of full-length LINC01468 and its deletion fragments of the SHIP2-binding domain in LINC01468. **G** SHIP2 pulled down by different LINC01468 constructs was tested by western blot. **H** The expression analysis of SHIP2 from 26 pairs of HCC tissues and corresponding para-cancerous tissues from human NAFLD-HCC by qRT-PCR. **I** Western blot analysis of SHIP2 in human NAFLD-associated HCC. Representative western blot images and expression levels of tumors (T) and adjacent nontumors (NT) from eight paired NAFLD-associated HCCs. **J** Pearson correlation analysis of LINC01468 and SHIP2 expression in 26 HCC tissues. **K**, **L** SHIP2 protein expression in palmitic acid- (**K**) or oleic acid-treated (**L**) SNU-182 cells (*n* = 3 per group). ^**^P < 0.01. **M**, **N** LINC01468 expression in (**M**) palmitic acid- or (**N**) oleic acid-treated SNU-182 cells by qRT-PCR (*n* = 3 per group). The data represent the mean ± S.D (*P < 0.05; **P < 0.01; ***P < 0.005).

Since most lncRNAs have been suggested to exert their actions by interacting with their counterpart proteins [29–32], we performed an RNA pull-down assay followed by mass spectrometry and western blot analysis to identify the proteins associated with LINC01468 (Fig. 4A). The Src homology 2 (SH2)-domain-containing PtdIns(3,4,5)P3 5-phosphatase-2 (SHIP2), which specifically hydrolyzes the phosphate at the 5ʹ position of the inositol ring to produce PtdIns(3,4)P2 from PtdIns(3,4,5)P3 [33], was the most-enriched LINC01468-interacting protein (Fig. 4B). Using biotin-LINC01468 pull-down lysates, we subsequently confirmed that LINC01468 and SHIP2 interacted in a dose-dependent manner (Fig. 4C). RNA immunoprecipitation (RIP) with an SHIP2 antibody was used to validate the association between LINC01468 and SHIP2. Notably, LINC01468 was enriched approximately 15-fold in precipitates with SHIP2 antibodies (Fig. 4D). A combination of fluorescence in situ hybridization (FISH) and immunofluorescence staining showed that endogenous LINC01468 was mainly colocalized with SHIP2 (Fig. 4E). We then determined the unique binding region of LINC01468 responsible for its interaction with SHIP2 and constructed a series of deletion mutants of LINC01468. RNA pull-down assays showed that LINC01468 mutants containing nucleotides 400–600 bound to SHIP2 as efficiently as full-length LINC01468, whereas other mutants completely lost their binding capacity, indicating that nucleotides 400–600 of LINC01468 are required for association with SHIP2 (Fig. 4F, G). Taken together, these results implied that LINC01468 directly interacts with SHIP2. Therefore, we performed expression analysis of SHIP2 from HCC tissues and para-cancerous tissues, which showed mRNA- (Fig. 4H) and protein-level (Fig. 4I) reductions in SHIP2 expression and a negative correlation between SHIP2 expression and the LINC01468 level in 26 paired tumors and adjacent normal tissues from human NAFLD-associated HCCs (Fig. 4J). SHIP2 was downregulated in the NAFLD cell model established using SNU-182 cells induced by palmitic acid (PA) and oleic acid (OA) (Fig. 4K–N). Thus, LINC01468 silencing inhibits HCC tumorigenesis via lipid metabolism.

### LINC01468 destabilizes SHIP2 via ubiquitin proteasome pathway

**Fig. 5:**
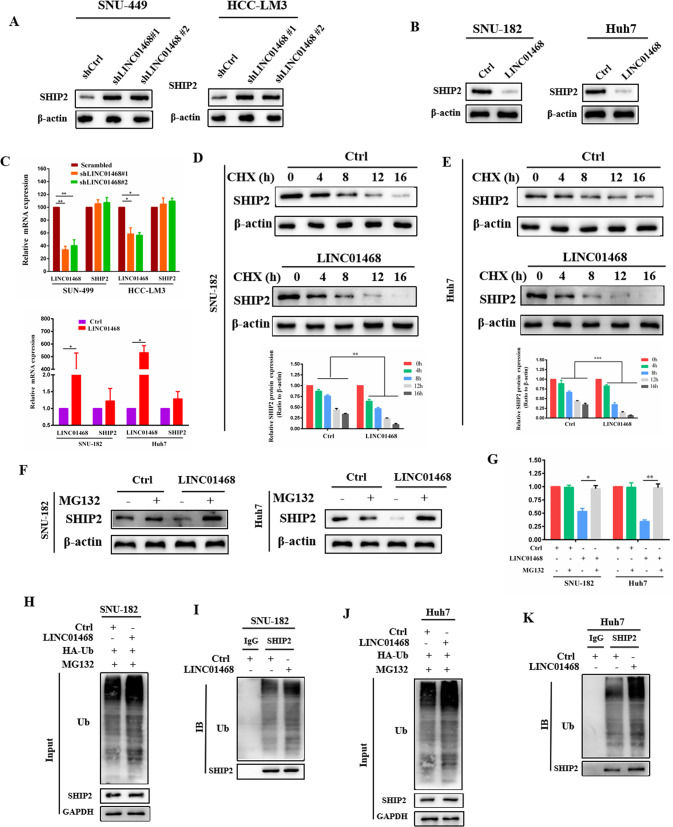
LINC01468 destabilizes SHIP2 via ubiquitin proteasome pathway. **A**, **B** Western blotting analysis of SHIP2 expression levels in SNU-449 and HCC-LM3 cells after silencing LINC01468 (**A**) or in SNU-182 and Huh7 cells after overexpression of LINC01468 (**B**). **C** RT-qPCR was used to test the mRNA expression of SHIP2 in indicated cells transfected with (upper panel) or without (lower panel) LINC01468. GAPDH was used for normalization. **D**, **E** SNU-182 (**D**) and Huh7 (**E**) transfected with or without LINC01468 were treated with CHX at 20 μg/mL for the indicated times, and the expression of SHIP2 was tested by western blotting. **F** SNU-182 and Huh7 transfected with or without LINC01468 were treated with MG132 (5 μM for 4 h), and the SHIP2 protein level was tested by western blotting. **G** Quantification of the results in **F. H** The LINC01468-overexpressing SNU-182 cells were cotransfected with HA-tagged ubiquitin (HA-Ub) expressed plasmid. After MG132 (5 μM for 4 h) treatment, cell lysates were subjected to western blotting. Anti-HA antibody was used to perform IP. **I** The cell lysates in **H** were subjected to Co-IP with the SHIP2 (IP: SHIP2) antibody followed by western blotting. **J** Western blotting was used to detect cell lysates from Huh7 cells cotransfected with HA-Ub plasmid with or without LINC01468 and treated with MG132 (5 μM for 4 h). **K** The cell lysates in **J** were subjected to Co-IP with SHIP2 antibody (IP: SHIP2) followed by western blot analysis. *P < 0.05; **P < 0.01; ***P < 0.005.

Since lncRNAs destabilize their binding proteins through ubiquitination-mediated degradation [34–36], we hypothesized that LINC01468 might bind to SHIP2 to regulate its stability. We found that LINC01468 silencing upregulated SHIP2 protein levels (Fig. 5A), whereas LINC01468 overexpression decreased SHIP2 protein levels in HCC cells (Fig. 5B). However, overexpression or silencing of LINC01468 had no effect on the SHIP2 mRNA (Fig. 5C). To determine whether LINC01468 regulates SHIP2 stability through ubiquitination-mediated degradation, we treated SNU-182 and Huh7 cells with the de novo protein synthesis inhibitor cycloheximide and the potent cell-permeable reversible proteasome inhibitor MG132, respectively. LINC01468 overexpression led to a robust decrease in SHIP2 protein levels (Fig. 5D, E), and MG132 rescued this reduction (Fig. 5F, G), suggesting that LINC01468 could promote SHIP2 for proteasome-dependent degradation. Furthermore, LINC01468 overexpression increased SHIP2 ubiquitination in both SNU-182 and Huh7 cells (Fig. 5H–K). Thus, LINC01468 can destabilize the SHIP2 protein by promoting its ubiquitination-mediated degradation.

### LINC01468 induces CUL4A binding to SHIP2 to promote SHIP2 ubiquitinated degradation

**Fig. 6:**
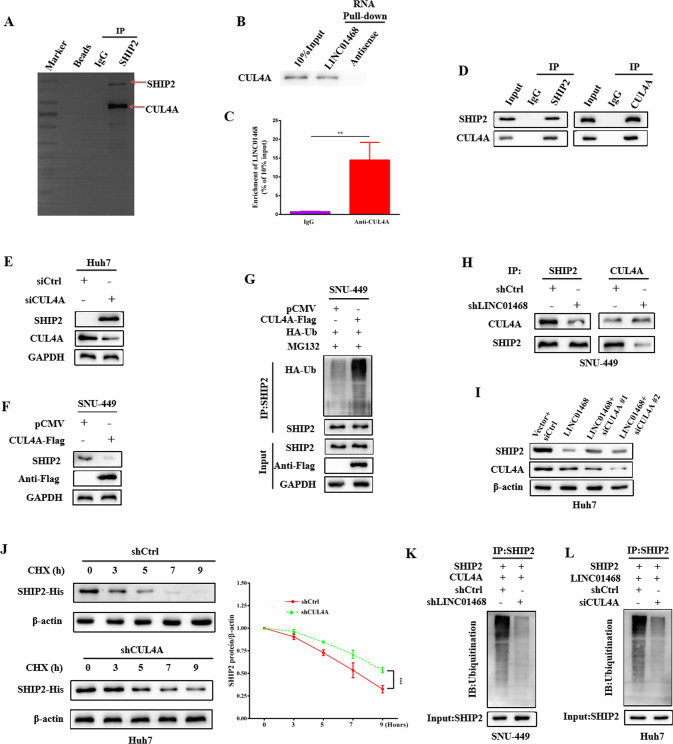
LINC01468 enhances binding of E3 ubiquitin ligase CUL4A to SHIP2. **A** After immunoblot analysis,the gel were stained with coomassie brilliant blue and the bands that were precipitated specifically by SHIP2 underwent mass spectrometric detection. **B** The binding of LINC01468 to CUL4A was revealed by LINC01468 pull-down followed by immunoblot analysis. **C** RIP assay showed that LINC01468 binds to CUL4A. The data represent the means ± S.D (**P < 0.01). **D** Co-IP showed the binding of SHIP2 to CUL4A. **E** Huh7 cells transfected with siCUL4A or **F** SNU-449 cells transfected with CUL4A-Flag plasmid were submitted to examine SHIP2 and CUL4A by western blotting. **G** CUL4A promotes SHIP2 ubiquitination. SNU-449 cells transfected with or without CUL4A-Flag plasmid were treated with MG132 (5 μM for 4 h), after which cells were subjected to Co-IP with SHIP2. **H** LINC01468 silencing decreased the interaction of SHIP2 with CUL4A. **I** Knockdown of CUL4A abrogated the LINC01468-mediated degradation of SHIP2 protein. **J** Silencing of CUL4A prolonged the degradation of SHIP2. Immunoblotting detection of His tag is shown. **K** Knockdown of LINC01468 reduced the ubiquitination of SHIP2. **L** Knockdown of CUL4A reduced the ubiquitination of SHIP2. The input of the cell lysates were showed in the bottom panels.

LncRNAs can participate in ubiquitin-mediated protein degradation by acting as scaffolds. To identify the E3 ubiquitin ligase targeting SHIP2 for degradation in HCC cells, we co-immunoprecipitated SHIP2 from the lysates of HCC cells and analyzed the immunoprecipitated proteins by liquid chromatography-mass spectrometry. CUL4A was identified as a candidate E3 ligase that binds to LINC01468, which mediates the ubiquitination of SHIP2 (Fig. 6A). RNA pull-down also revealed the interaction of LINC01468 with CUL4A (Fig. 6B). RNA-immunprecipitation (RIP) assays followed by qRT-PCR validated that LINC01468 was markedly enriched in the RNA-protein complexes precipitated with the anti-CUL4A antibody (Fig. 6C). We then validated the interaction between endogenous SHIP2 and CUL4A in HCC cells by immunoprecipitation (Fig. 6D). Importantly, CUL4A silencing increased the level of SHIP2 protein (Fig. 6E), whereas CUL4A overexpression reduced SHIP2 protein levels (Fig. 6F). As expected, CUL4A overexpression increased SHIP2 ubiquitination (Fig. 6G). Next, we examined whether LINC01468 affected the SHIP2-CUL4A interaction. We found that LINC01468 silencing markedly decreased the interaction of SHIP2 with CUL4A (Fig. 6H). To confirm whether SHIP2 degradation is mediated by CUL4A, we silenced CUL4A and detected the SHIP2 protein level, and showed that CUL4A silencing decreased LINC01468-dependent SHIP2 degradation (Fig. 6I). The degradation assay showed that the half-life of SHIP2 was prolonged (Fig. 6J). Moreover, LINC01468 or CUL4A silencing dramatically reduced SHIP2 ubiquitination (Figs. 6K and 4L). Thus, CUL4A is an E3 ligase that regulates SHIP2 ubiquitination.

### LINC01468 as a potential therapeutic target for drug resistance in HCC

**Fig. 7:**
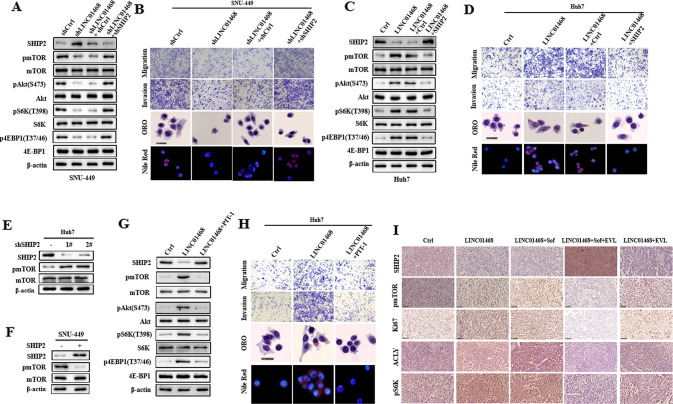
Enforced expression of SHIP2 rescues the LINC01468 metabolic phenotypes. **A** Western blotting analysis of the SHIP2/Akt/mTOR pathway protein in SNU-449 cells cotransfected with or without shLINC01468, together with shSHIP2 or empty vector. **B** Migration, invasion, ORO staining, and Nile Red staining were performed in SNU-449 cells as described in **A**. **C** Western blotting analysis of the SHIP2/Akt/mTOR pathway protein in Huh7 cells cotransfected with LINC01468 or the control, together with SHIP2 or empty vector. **D** The migration, invasion, ORO staining, and Nile red staining were performed in Huh7 cells as described in **B**. **E**, **F** Immunoblot detection of pmTOR level in SHIP2 **E** silencing in Huh7 cells or **F** overexpression in SNU-449 cells. **G** Immunoblotting of the indicated protein lysates from Huh7 cells expressing Ctrl or LINC01468 treated with or without the PIP3 inhibitor PIT-1. **H** Migration, invasion, ORO staining, and Nile Red staining were performed in SNU-449 cells as described in **G**. **I** Expression of LINC01468/SHIP2/mTOR cascade and Ki67 in HCC xenograft tissues. The expression of SHIP2, pmTOR, p-4E-BP1, and Ki67 was examined by IHC. Scale bars, 50 μm. Sof sorafenib, EVL everolimus (the mTOR inhibitor).

Although SHIP2 can suppress PI3K/Akt signaling and inhibits cancer progression [37–39], its role in regulating the PI3K/AKT/mTOR signaling pathway in HCC remains poorly understood. We found that LINC01468 silencing decreased the levels of phosphorylated AKT (S473), phosphorylated mTOR, phosphorylated S6K, and 4EBP1, which recovered after SHIP2 knockdown (Fig. 7A). Consistent with the changes in the expression of these Akt/mTOR proteins, we silenced SHIP2 in LINC01468-knockdown cells and confirmed that LINC01468-mediated metabolic regulation is indeed channeled through SHIP2 (Fig. 7B). Accordingly, the LNC04168-knockdown-induced reduction in tumor growth was reversed by SHIP2 knockdown in a SNU-449 HCC model stably transfected with an shRNA for LNC04168 in vivo, suggesting that LNC04168 acts through SHIP2 downregulation to promote the growth of HCC tumors (Supplementary Fig. 2A and B). Conversely, LINC01468 overexpression increased Akt and mTOR levels, whereas SHIP2 overexpression abolished LINC01468-induced activation of PI3K/AKT/mTOR signaling (Fig. 7C). In accordance with these changes, we confirmed that LINC01468-mediated metabolic regulation is channeled through SHIP2 after ectopic expression of SHIP2 in LINC01468-overexpression cells (Fig. 7D). We also found that SHIP2 silencing led to an increased level of mTOR protein (Fig. 7E), and enforced expression of SHIP2 decreased mTOR protein (Fig. 7F), indicating that SHIP2 negatively regulates PI3K/Akt signaling in HCC. Taken together, these data suggest that the LINC01468/SHIP2 axis activates the PI3K/AKT/mTOR signaling pathway. To confirm that the LINC01468-mediated metabolic regulation is channeled through SHIP2/ phosphatidylinositol-3,4,5 -trisphosphate (PIP3), we used the PIP3 inhibitor PIT-1 in LINC01468-overexpressed cells. PIT-1, a small molecule PIP3 antagonist (PIT) that blocks pleckstrin homology (PH) domain interaction, including activation of Akt, significantly inhibits tumor angiogenesis and metastasis [40, 41]. PIT-1 was able to inhibit the LINC01468 overexpression induced SHIP2/PIP3-dependant activation of Akt/mTOR (Fig. 7G) and rescue LINC01468-induced metabolic phenotypes. The rescued phenotypes included a lower ability for migration and invasion and decreased lipid production (Fig. 7H). Thus, SHIP2/PIP3 are the effectors of LINC01468 in modulating lipid metabolism. The expression of LINC01468, SHIP2, and mTOR pathways was confirmed in the xenograft by IHC, and Ki67 staining indicated cell proliferation in these tumors (Fig. 7I). Therefore, LINC01468 is a potential therapeutic target for HCC and drug resistance.

### m^6^A modification mediated by ALKBH5 upregulated LINC01468

**Fig. 8:**
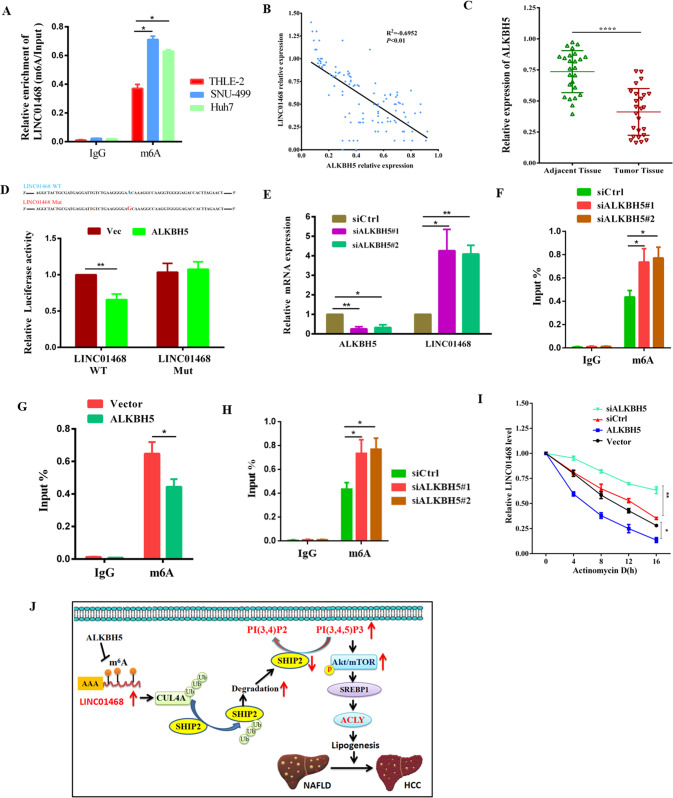
m^6^A modification mediated by ALKBH5 upregulates LINC01468. **A** The enrichment of m^6^A-modified LINC01468 was detected by the RIP-RT-qPCR assay in the indicated cells. **B** The negative correlation between the levels of LINC01468 and ALKBH5 was confirmed in 26 pairs of NAFLD-HCC patients. **C** ALKBH5 expression in HCC was analyzed in HCC tissues and corresponding para-cancerous tissues from 26 pairs of NAFLD-HCC patients. **D** The online website http://m6avar.renlab.org/ was used to predict the m^6^A modification locus of LINC01468 modified by ALKBH5, followed by confirmation by the luciferase reporter assay. **E**, **F** Effect of ALKBH5 overexpression in SNU-449 cells (**E**) or knockdown in Huh7 cells (**F**) on LINC01468 expression. **G**, **H** Results of RIP-RT-qPCR showing the enrichment of m^6^A-modified LINC01468 following overexpression in SNU-449 cells or silencing in Huh7 cells of ALKBH5. **I** LINC01468 stability was analyzed in HCC-LM3 cells with ALKBH5 overexpression or silencing in the presence of actinomycin (**D**). The data represent the means ± S.D (*P < 0.05; **P < 0.01; ****P < 0.001). **J** A schematic model for a positive feedback loop between LINC01468 and NAFLD-associated hepatocellular carcinoma. NAFLD upregulated the lipogenesis-related lncRNA, LINC01468, which serves as a molecular glue to bind the E3 ligase CUL4A to SHIP2 and induce SHIP2 degradation through ubiquitination. The reduction in SHIP2 protein results in the accumulation of PIP3, recruiting AKT to the plasma membrane, where AKT/mTOR is activated to facilitate lipogenesis and HCC progression.

Since m^6^A dysregulation enhances lipogenesis and NAFLD-HCC progression [42], we analyzed whether LINC01468 was modified or upregulated by m^6^A modification. Many m^6^A sites were found with LINC01468 using the RMvar (rmvar.renlab.org) prediction. In comparison with normal THLE2 liver cells, m^6^A was more significantly abundant in Huh7 and SNU-449 cells in RIP and RT-qPCR results (Fig. 8A). To screen the m^6^A enzyme-regulated LINC01468 modification, antibodies against different m^6^A-related proteins were used to perform an RIP assay and detect the expression of LINC01468 in the pulled products. METTL3 and ALKBH5 significantly enriched LINC01468, suggesting that METTL3 and ALKBH5 play roles in m^6^A modification of LINC01468. Interestingly, ALKBH5 expression was negatively correlated with LINC01468 expression in HCC (Fig. 8B). In comparison with para-cancerous tissues, HCC tissues showed significantly reduced ALKBH5 levels (Fig. 8C). Further experiments validated that site 52455230 could be modified by ALKBH5. ALKBH5 overexpression led to increased luciferase activity in the wild-type LINC01468 group, whereas luciferase activity was unchanged in the mutant-type LINC01468 group (Fig. 8D). ALKBH5 overexpression decreased LINC01468 mRNA expression in HCC cells (Fig. 8E), whereas ALKBH5 silencing had the opposite result (Fig. 8F). RIP qPCR assays showed that ALKBH5 overexpression reduced the m^6^A modification of LINC01468 in HCC cells (Fig. 8G), whereas ALKBH5 silencing produced the opposite effect (Fig. 8H). In the presence of actinomycin D, an inhibitor of de novo synthesis of RNA, ALKBH5 overexpression decreased the stability of LINC01468, whereas ALKBH5 silencing showed the opposite result (Fig. 8I). These data reveal the critical role of ALKBH5 in upregulating LINC01468 in HCC.

## Discussion

The carcinogenic pathways leading to HCC tumorigenesis in NAFLD patients are complex and poorly understood. Epigenetics has been implicated in the etiology of NAFLD-associated HCC [43, 44], and the role of lncRNAs in several NAFLD-associated cancer-related processes participating in HCC tumorigenesis, such as epigenetic regulation and cell metabolism, has received much attention [45]. Although some lncRNAs may contribute to NAFLD-HCC progression [46–48], their role in NAFLD-associated HCC is largely unclear. The present study investigated the role of LINC01468 in the progression of NAFLD-HCC and showed that LINC01468 mediates lipogenesis, thereby promoting HCC progression through CUL4A-linked degradation of SHIP2 (Fig. 8J). Many lncRNAs are dysregulated in HCC and play critical roles in tumorigenesis and HCC progression [49, 50], and some HCC-related lncRNAs play crucial roles in the initiation and progression of HCC by regulating lipid metabolic reprogramming [51–54]. In examining the role of lncRNAs in NAFLD-associated HCC, the authors found that LINC01468 was upregulated in liver tissues during NAFLD-HCC and that LINC01468 silencing inhibited HCC tumorigenesis via lipid metabolism. Since lncRNAs have been shown to mediate resistance to treatment and malignant progression of HCC [55, 56], these results suggest that LINC01468 promotes HCC proliferation and confers drug chemoresistance in HCC cells. Thus, we uncovered a new role of LINC01468 in HCC development.

Certain lncRNAs function biologically by interacting with other proteins [29–32], while others regulate their binding proteins through post-translational modifications. To identify the molecular mechanisms underlying the oncogenic role of LINC01468 in HCC, an RNA pull-down assay and western blot analysis were used to determine whether SHIP2 is associated with LINC01468. RNA-IP was used to validate the association between LINC01468 and SHIP2. Since LncRNAs destabilize their binding proteins by promoting ubiquitination-mediated degradation [34–36], we postulated that LINC01468 might bind to SHIP2 to regulate its stability. Our results showed that in HCC cells, LINC01468 silencing upregulated SHIP2 protein levels, and LINC01468 overexpression decreased SHIP2 protein levels, which were rescued by MG132; thus, LINC01468 could promote SHIP2 for proteasome-dependent degradation. Additionally, mechanistic details relating to the ability of LINC01468 to regulate SHIP2 suggested that LINC01468 promotes SHIP2 ubiquitination by enhancing its binding to CUL4A, a ubiquitin E3 ligase, thereby leading to CUL4A-dependent SHIP2 ubiquitinated degradation.

SHIP2 regulates the PI3K/AKT pathway, which plays a crucial role in cancer progression, by producing PI(3,4)P2 to increase AKT activation and cancer cell survival. SHIP2 plays a central role in cancer development and progression, including HCC [57, 58], and SHIP2 has been shown to negatively regulate PI3K/Akt signaling and suppresses cancer progression [37–39]. Mechanistic details related to SHIP2 modulation may involve proteasome-dependent degradation, and a recent study showed that S-phase kinase-associated protein 2 (SKP2), a component of the E3 ubiquitin ligase complex, downregulates SHIP2 through polyubiquitination. Our results confirmed that LINC01468 increases CUL4A-mediated degradation of SHIP2, by which SHIP2 negatively regulates PI3K/Akt, thereby promoting lipogenesis and HCC progression; thus, SHIP2 functions as a tumor suppressor in NAFLD-HCC. Moreover, owing to its ability to produce PI(3,4)P2, SHIP2 can actually promote Akt activation, and SHIP2 inhibition can kill breast and colon cancer cells; thus, SHIP2 may function as an oncogene as well [59, 60]. However, the role of SHIP2 in different tumors remains to be determined.

Taken together, the present results reveal a new mechanism by which LINC01468-mediated lipogenesis promotes NAFLD-HCC progression through the CUL4A-linked degradation of SHIP2. LINC01468 acts as a crucial driver of NAFLD-HCC progression and chemoresistance, highlighting the value of the LINC01468-SHIP2 axis as a potential therapeutic target for HCC.

## Materials and methods

### Cell culture

Normal human hepatocyte THLE2 and the HCC cell lines (Huh7, SNU-449, SNU-182, and HCC-LM3) were obtained from the American Type Culture Collection (ATCC, USA) (Supplementary Table 1).

### Patients and clinical samples

The clinical tumor and adjacent matched non-tumor tissues were collected from patients with NAFLD-HCC (*n* = 26) at the First Affiliated Hospital of Guangxi Medical University (Table 1). All studies involving human samples were reviewed and approved by the ethics committee of the First Affiliated Hospital of Guangxi Medical University, and written informed consent was obtained from all patients based on the Declaration of Helsinki. pTNM classification advocated by the International Union against Cancer was uused to determine tumor grade and classification.

### Further applied methods

CCK8 assay, Reverse transcription quantitative polymerase chain reaction (RT-qPCR),cell proliferation assay and drug treatment, Immunohistochemistry (IHC), methylated RNA immunoprecipitation qPCR (MeRIP-qPCR), xenograft assay, RNA immunoprecipitation (RIP) assay, RNA pull-down assay, Histological analysis for lipid droplet determination, triglyceride and cholesterol assay, m^6^A quantification, coimmunoprecipitation, and western blot analysis, RNA fuorescence in situ hybridization (RNA-FISH) assay are further described in Supplementary materials and methods.

### Statistical analysis

Statistical analysis was conducted in the GraphPad Prism v8.0 (GraphPad, Inc., USA) and the Statistical Software Package for Social Sciences (v 22.0; SPSS, Inc., Chicago, IL, USA). Differences were considered statistically significant at *P* < 0.05. Pearson’s correlation analysis was fitted between two selected genes in clinical tumor tissues.

## Supplementary information

Supplementary Figures

Supplementary materials and methods

Supplementary Tables

Unprocessed WB

## Data Availability

The data used to support the findings of this study are available from the corresponding author upon request.
