# Supplementary material for: LINC01468 drives NAFLD-HCC progression through CUL4A-linked degradation of SHIP2
Source: Cell Death Discov. 2022 Nov 7;8:449. doi: 10.1038/s41420-022-01234-8 (PMC9640567; doi:10.1038/s41420-022-01234-8)
Supplement: Supplementary file 1 — Supplementary Figures [file 41420_2022_1234_MOESM1_ESM.doc]

**
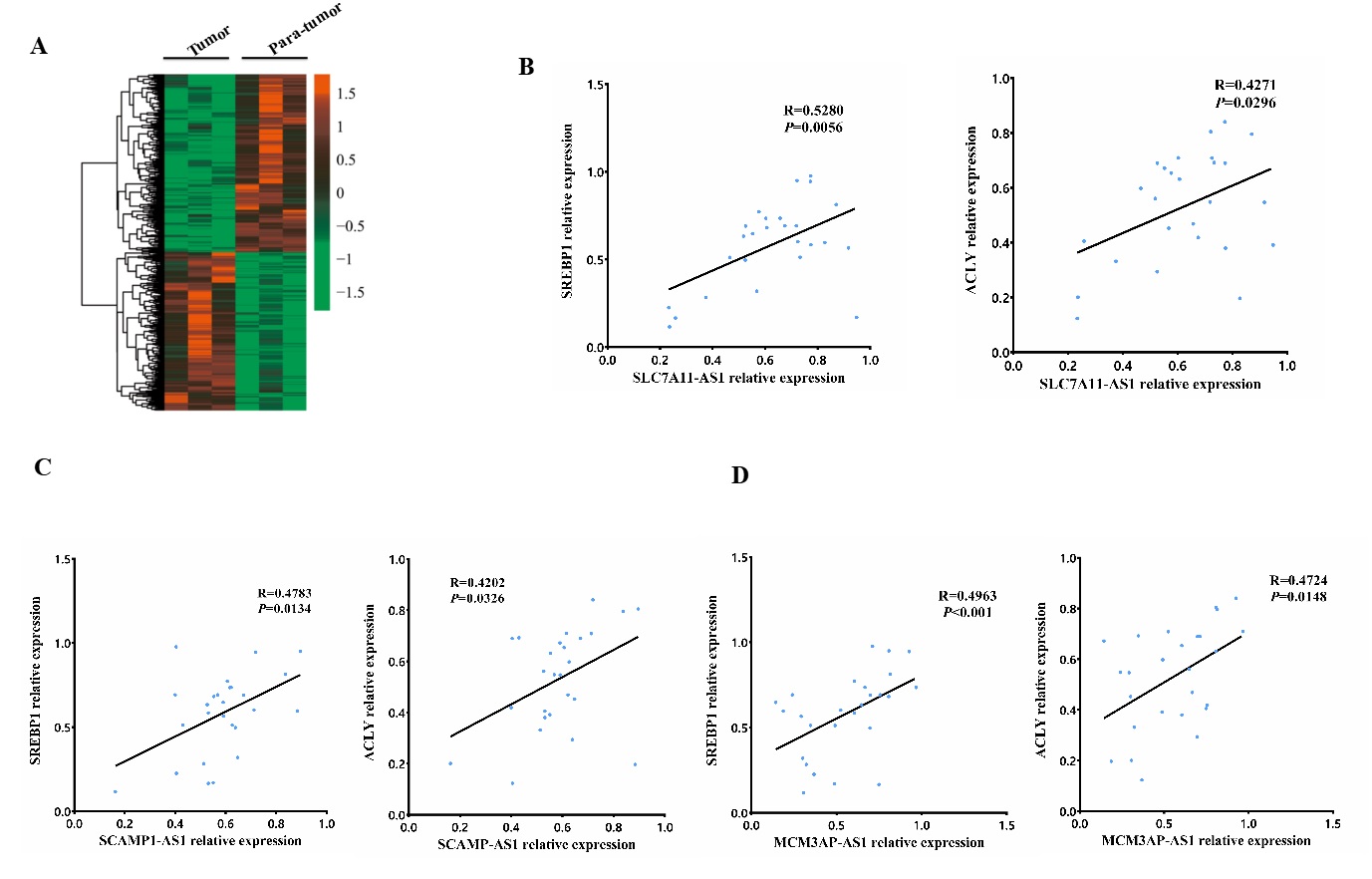
Supplementary Fig 1.** (A) Heatmap summarizing RNA-Seq data for mice with NAFLD-HCC. *P*<1.78×10^–9^. (B-D) Scatter plot analysis of correlation between mRNA levels of indicated LncRNA and SREBP1 or ACLY in 26 HCC tissues.

**
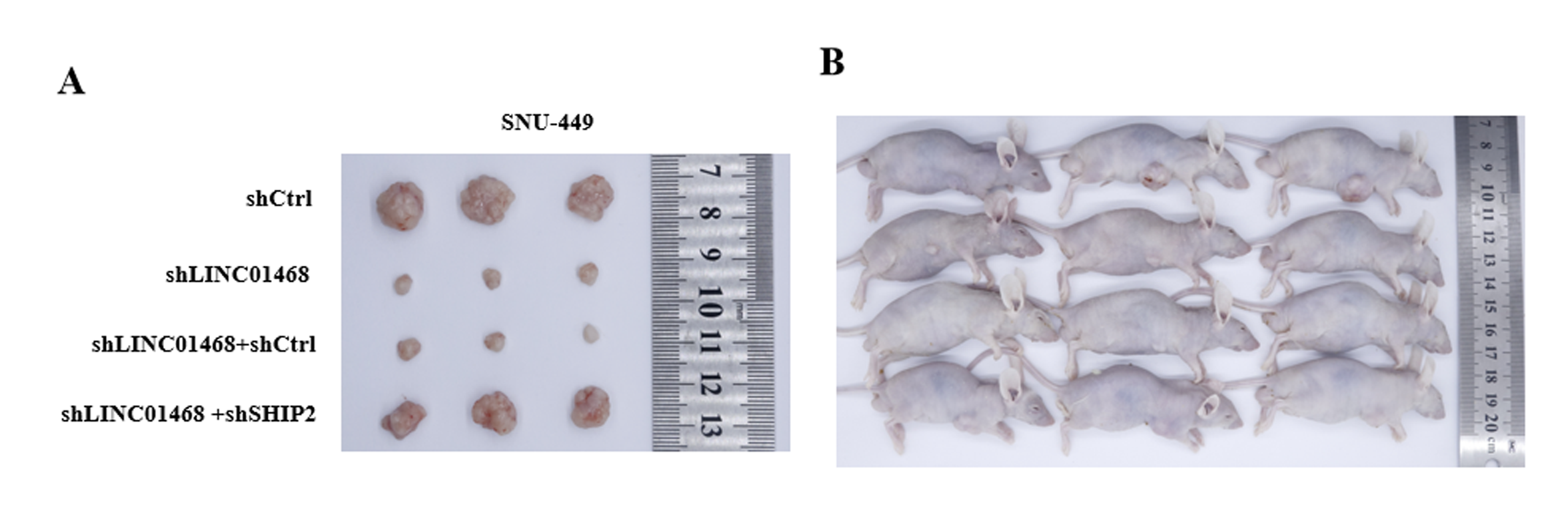
**

**Supplementary Fig 2.** (A) Representative tumor images of xenografts derived from SNU-449 cells stably transfected with indicated shRNA. (B) The right flanks of all the mice were subcutaneously injected with 5 × 10^6^ cells. The tumors were collected after 4 weeks.
