# Supplementary material for: LINC01468 drives NAFLD-HCC progression through CUL4A-linked degradation of SHIP2
Source: Cell Death Discov. 2022 Nov 7;8:449. doi: 10.1038/s41420-022-01234-8 (PMC9640567; doi:10.1038/s41420-022-01234-8)
Supplement: Supplementary file 2 — Supplementary materials and methods [file 41420_2022_1234_MOESM2_ESM.doc]

**Supplementary materials and methods**

**NAFLD-promoted HCC mouse models**

The obesity-promoted HCC models were constructed as described previously[1]. For the dietary NAFLD-HCC model, DEN-treated C57BL/6 mice (6-week-old) were separated into two dietary groups and fed either low-fat diet (LFD, composed of 12% fat, 23% protein, 65% carbohydrates based on caloric content) or high-fat diet (HFD, composed of 60% fat, 15% protein, 25% carbohydrates based on caloric content).

**Generation of subcutaneous xenograft mouse model**

Four-week-old BALB/c nude mice were purchased from the Animal Center of Guangxi Medical University (Nanning, China). All animal experimental protocols were performed in accordance with the National Institutes of Health Animal Use Guidelines on the Use of Experimental Animals. HCC-LM3 cells with silenced LINC01468 or the negative control were subcutaneously injected into the flank region of the mice at 2 × 10^6^cells. Tumor volume was measured weekly and calculated as follows: *V*=(length×width^2^)/2. After five weeks, the tumors were excised and weighed.

**RNA sequencing (RNA-seq)**

Total RNA was extracted from NAFLD-promoted HCC mouse models shNC and shLINC01468 groups of SNU-449 cells using ESscience RNA-Quick Purification Kit (YiShan Biotech, Shanghai, China), and each group was prepared with three parallel replicates. Then a total of 6 samples were sent to BoAo Corporation (China) for further RNA-seq detection and analysis. Differentially expressed genes (DEGs) between two groups were identified by DEGseq method. The pathway analysis for DEGs was performed based on the KEGG database.

**Reverse transcription quantitative polymerase chain reaction (RT-qPCR)**

Total RNA was extracted usingan RNA simple Total RNA Kit (TIANGEN, DP419) according to the manufacturer’s instructions. Complementary DNA (cDNA)was synthesized using a RevertAid First Strand cDNA Synthesis Kit (Thermo Scientific,#K1622) and poly(A) polymerase reaction buffer (NEB,M0276s) according to the manufacturer’s instructions. RT-qPCRwas performed using the iTaqTM Universal SYBR®Green Supermix. The fold-change in RNA expression was quantified using the 2^-ΔΔCt^method. The primer sequences used in this study are listed in Supplementary table 2.

**Cell proliferation assay and drug treatment**

Transfected cells (1×10^3^) were seeded in 96-well plates and cell viability was detected every 24 hours for 5 days using a Cell Counting Kit-8 (CCK-8) assay (Dojindo). For the colony formation assay, transfected cells were seeded in 6-well plates at a density of 400 cells per well, and incubated for approximately 10 days. The cells were then fixed, stained, and counted. In addition, 1×10^3^ cells were incubated with lenvatinib or sorafenib for 72 hours and then subjected to a CCK-8 assay. For the clonogenic survival assay, 800 cells were treated with lenvatinib or sorafenib with indicated dose for 2 hours and then cultured for approximately 10 days.

**Immunohistochemistry (IHC)**

IHC was performed on paraformaldehyde-fixed, paraffin-embedded tissue specimens using heat-mediated antigen retrieval citrate (0.01 M, pH6.0). Endogenous peroxidase activity was blocked using 3% H_2_O_2_ for 15 min at room temperature (RT). Thereafter, the sections were incubated with goat serum for 1 h to block the non-specific binding sites andthen with primary antibodies overnight at 4℃. After rinsing three times for 5 min each with PBS, the sections were further incubated with horseradish peroxidase-conjugated secondary antibodies for 1 h at RT. Each section was then rinsed with PBS thrice for 5 min each, and the reactions were developed using diaminobenzidine tetrahydrochloride (DAB) as a substrate. Cellular nuclei were counterstained using hematoxylin, and the sections were sealed with neutral gum.Images were obtained using an Olympus X71 inverted microscope(Olympus Corp., Tokyo, Japan).

**Methylated RNA immunoprecipitation qPCR (MeRIP-qPCR)**

m6A-RNA immunoprecipitation (Me-RIP) was performed as previously described [2]**.** The MeRIP-qPCR assay was performed to determine the level of LINC01468 m^6^A. Total intracellular RNA was extracted using TRIzol reagent. Anti-m^6^A antibodies or anti-immunoglobulin G (IgG; Cell Signaling Technology) (3 μg) was first conjugated to protein A/G magnetic beads and mixed with 100 μg aliquot of total RNA in IP buffer containing RNase/protease inhibitors.m^6^A-modified RNA was eluted twice with 6.7 mM N^6^-methyladenosine 5'-monophosphate sodium salt at 4 ºC for 1 h. Subsequently, RT-qPCR analysis was performed to determine the m^6^A enrichment on LINC01468.

**RNA immunoprecipitation (RIP) assay**

A Magna RIP™ RNA-Binding Protein Immunoprecipitation Kit (Millipore, USA) was used according to the manufacturer’s instructions[3]. Briefly, cell extracts were immunoprecipitated with sepharose beads conjugated antibodies against AGO2 or IgG at 4 °C for 6 h. To remove proteins from the complex, 0.1% SDS/Proteinase K (0.5 mg/mL, 30 min at 55°C) was used. Immunoprecipitated proteins and RNAs were detected using western blot and RT-qPCR, respectively.

**RNA pull-down assay**

A Pierce Magnetic RNA-Protein Pull-Down Kit (Thermo Fisher Scientific,20164) was used according to the manufacturer’s instructions. In brief, cell lysates were treated with RNAase-free DNAase I and incubated with biotinylated LINC01468 in the presence of streptavidin magnetic beads, which can capture the proteins/miRNAs potentially interacting with LINC01468. A Pierce™ RNA 3' End Desthiobiotinylation Kit (Thermo,20163) was used for LINC01468 biotinylation labeling. Proteins and RNAs in the captured protein-RNA complex were analyzed using western blotting and mass spectrometry, respectively.

**Histological analysis for lipid droplet determination**

The accumulation of lipid droplets was observed by H&E and Oil Red O staining[4]. Following anaesthesia (ketamine 80 mg/kg and xylazine 12 mg/kg, i.p), mouse livers were excised and immediately placed in 10% neutral-buffered formalin at room temperature for 24 h after a brief rinse with PBS. The tissues were dehydrated through serial alcohols and cleared in xylenes. The specimen were embedded in paraffin, cut in 5 µm sections and stained with haematoxylin and eosin (H&E). For Oil Red O staining, livers were sliced and snap-frozen in isopentane-cooled liquid nitrogen prior to cutting into 10 µm sections with a cryostat. Sections were fixed with 4% paraformaldehyde and placed in absolute propylene glycol for 5 min, then stained in pre-warmed Oil Red O solution for 15 min at 60 ℃ followed by the differentiation with 85% propylene glycol and brief counterstaining. Sections were then mounted with aqueous VECTASHIELD mountant (Vector Laboratories Ltd, Burlingame, CA). A digital Olympus BX-51 microscope (400×) (Olympus America Inc., Melville, NY) was used to digitalize sections. Quantification of lipid droplets (average size and fraction) measured by H&E and Oil Red O staining in each group were calculated using the colour-based threshold plugin of the ImageJ (version1.43u, NIH) software.

**Triglyceride and cholesterol assay**

Cells were washed twice with PBS and lysed[5]. Total protein concentration was detected using the bicinchoninic acid assay kit (Abcam). Intracellular triglyceride and cholesterol were measured by using the triglyceride assay kit (Biovision, USA) and cholesterol assay kit (Biovision, USA) according to the protocol.

**m^6^A quantification**

The mRNA global m6A levels in the OS tissue and cells were detected using the EpiQuik m6A RNA Methylation Quantification Kit (Epigentek, Colorimetric) following the manufacturer’s pro­tocol. 200 ng poly-A-purified RNA was used for each sample analysis.

**Coimmunoprecipitation**

Cells were collected in cold PBS and lysed with RIPA buffer (50 mM Tris (pH 7.4), 150 mM NaCl, 1 mM EDTA, 0.1% SDS, 1% Nonidet P-40 (NP-40), 0.5% sodium deoxycholate, 0.5 mM DTT, and protease inhibitor)[3]. The lysates were diluted 2- to 4-fold with dilution buffer (50 mM Tris (pH 7.4), 100 mM NaCl, 1 mM EDTA, 0.1% NP-40, 10% glycerol, and protease inhibitor). Then, 2-5 μg of antibodies were added to the diluted cell lysates, and the mixtures were incubated overnight at 4°C. The next day, the protein complexes were isolated with magnetic Protein G Dynabeads for 2 h at 4°C with rotation. The bead–antibody–protein complexes were then washed 4 times with wash buffer (50 mM Tris (pH 7.4), 125 mM NaCl, 1 mM EDTA and 0.1% NP-40) and boiled for Western blot analysis.

**Western blot analysis**

Tissues or cells were homogenized and lysed with lysis buffer (50 mM Tris-HCl, 137 mM NaCl, 10% glycerol, 100 mM sodium orthovanadate, 1 mM phenylmethylsulfonyl fluoride (PMSF), 10 mg/ml aprotinin, 10 mg/ml leupeptin, 1% NP-40, and 5 mM protease inhibitor cocktail; pH 7.4). After protein concentration determination using a bicinchoninic acid (BCA) assay, β-mercaptoethanol and bromophenol blue were added to the sample buffer for electrophoresis. The proteins were separated via 10% PAGE and transferred to polyvinylidene difluoride membranes (Bio-Rad, Shanghai, China). The membranes were incubated with primary antibodies overnight at 4°C(Supplementary Table 3). After incubation with secondary antibodies for 2 h, the reactive bands were visualized using an enhanced chemiluminescence system. The intensities of the bands were quantified using an image analysis system[3].

REFERENCES

1. Tian Y, Wong VW, Wong GL, Yang W, Sun H, Shen J, et al. Histone Deacetylase HDAC8 Promotes Insulin Resistance and β-Catenin Activation in NAFLD-Associated Hepatocellular Carcinoma. Cancer Res. 2015;75:4803-16.

2. Chen Y, Peng C, Chen J, Chen D, Yang B, He B, Hu W, Zhang Y, Liu H, Dai L, et al. WTAP facilitates progression of hepatocellular carcinoma via m6A-HuR-dependent epigenetic silencing of ETS1. Mol Cancer. 2019;18:127.

3. Zhang Y, Kang M, Zhang B, Meng F, Song J, Kaneko H, et al. m(6)A modification-mediated CBX8 induction regulates stemness and chemosensitivity of colon cancer via upregulation of LGR5. Mol Cancer. 2019;18:185.

4. Guo R, Xu X, Babcock SA, Zhang Y, Ren J. Aldehyde dedydrogenase-2 plays a beneficial role in ameliorating chronic alcohol-induced hepatic steatosis and inflammation through regulation of autophagy. J Hepatol. 2015;62:647-56.

5. Wen J, Min X, Shen M, Hua Q, Han Y, Zhao L, Liu L, Huang G, Liu J, Zhao X. ACLY facilitates colon cancer cell metastasis by CTNNB1. J Exp Clin Cancer Res. 2019;38:401.
