# Supplementary material for: LINC01468 drives NAFLD-HCC progression through CUL4A-linked degradation of SHIP2
Source: Cell Death Discov. 2022 Nov 7;8:449. doi: 10.1038/s41420-022-01234-8 (PMC9640567; doi:10.1038/s41420-022-01234-8)
Supplement: Supplementary file 3 — Supplementary Tables [file 41420_2022_1234_MOESM3_ESM.doc]

**Supplementary Tables**

**Supplementary Table 1: Cell lines**

| Name | Source | Cat # |
| --- | --- | --- |
| THLE2 | ATCC | HTX1939 |
| SNU449 | ATCC | CRL-2234™ |
| HCC-LM3 | ATCC | AC338460 |
| Huh7 | ATCC | Y-0105 |
| SNU182 | ATCC | CRL-2235™ |

**Supplemental Table 2: Primers for qRT-PCR**

| Primer | Sequence(5’to 3’) |
| --- | --- |
| SLC7A11-AS1-Forward | 5′-AGCCTGGGTGATAAAGTG-3′ |
| SLC7A11-AS1-Reverse | 5′-TAAGCCCTCAATGGATAG-3′ |
| SCAMP1-AS1-Forward | 5′-GCGAAGGAAACGGGAACTGA-3′ |
| SCAMP1-AS1-Reverse | 5′-CAAGGCTGCTCTCCGTGATT-3′ |
| LINC01468-Forward | 5′-CCAGGGCTAGAGTAAGAGCC-3′ |
| LINC01468-Reverse | 5′-ACTGGAGTTGTCTCAGTGTAGG-3′ |
| MCM3AP-AS1-Forward | 5′-GCTGCTAATGGCAACACTGA-3′ |
| MCM3AP-AS1-Reverse | 5′-AGGTGCTGTCTGGTGGAGAT-3′ |
| SREBP1-Forward | 5′-GGAGCCATGGATTGCACATT-3′ |
| SREBP1-Reverse | 5′-CAGGAAGGCTTCCAGAGAGG-3′ |
| ACLY-Forward | 5′-ACGCCCCTGAAGACAAGAAA-3′ |
| ACLY-Reverse | 5′-CAAGGGGATTGATCTCGAGG-3′ |
| FASN-Forward | 5′-TCATCCGCTCGTTGTACCAGT-3′ |
| FASN-Reverse | 5′-TGGACTTGGTGGAGCCGAT-3′ |
| SCD1-Forward | 5′-CACTTGGGAGCCCTGTATGG-3′ |
| SCD1-Reverse | 5′-TGAGCTCCTGCTGTTATGCC-3′ |
| ACAC-Forward | 5′-CATCTCCACCCCTGTTGCAG-3′ |
| ACAC-Reverse | 5′-TCCAAAAAGACCTAGCCCTCAA-3′ |

**Supplementary Table 3: Primary antibodies for western blot**

| Reagent or resource | Source | Cat # |
| --- | --- | --- |
| Anti-p-mTOR S2481-antibody | Abcam | ab232486 |
| Anti-mTOR-antibody | Abcam | ab2833 |
| Anti-p-S6K-antibody | Abcam | ab9974 |
| Anti-S6K-antibody | Abcam | ab9366 |
| Anti-p-ACC S79-antibody | Abcam | ab222774 |
| Anti-ACC-antibody | CST | 3676 |
| Anti-SREBP1-antibody | Abcam | ab28481 |
| Anti-β-actin-antibody | CST | 3700 |
| Anti-p-4E-BP1-antibody | CST | 13443 |
| Anti-4E-BP1-antibody | CST | 9452 |
| Anti-SHIP2-antibody | proteintech | 20145-1-AP |
| Anti-FASN-antibody | Abcam | Ab128856 |
| Anti-ACLY-antibody | Abcam | ab40793 |
| Anti-SCD1-antibody | Abcam | ab236868 |
| Anti-GAPDH-antibody | proteintech | 60004-1-Ig |

**Supplementary Table 4：Plasmids for transfection in this study**

| Name | Sequence (5’to 3’) |
| --- | --- |
| shLINC01468#1 | CCGGGCAGTGCAGGTTAATTCAATGCTCGAGCATTGAATTAACCTGCACTGCTTTTTT |
| shLINC01468#2 | CCGGGCAGGTTAATTCAATGCCAGACTCGAGTCTGGCATTGAATTAACCTGCTTTTTT |
| shLINC01468#3 | CCGGGGTGTATATTCAGTGTGAACACTCGAGTGTTCACACTGAATATACACCTTTTTT |
| shLINC01468#4 | CCGGGATCTAATCGCAGTGAAATATCTCGAGATATTTCACTGCGATTAGATCTTTTTT |
| siCUL4A#1 | GCCUAGAGCUGUUUAGGAATT |
| siCUL4A#2 | GCGAGUACAUCAAGACCUUTT |
| siCUL4A#3 | GCUGCUAUAGUCAGAAUAATT |

**Supplementary Table 5:** **The reagents or resource for cell proliferation, invasion, motility, confocal analysis.**

| Reagent or resource | Source | Cat # |
| --- | --- | --- |
| Biocoat Matrigel Invasion Chambers | Corning | #354480 |
| Cell Culture insert with 8.0mm Membrane | Falcon | # 353097 |
| CCK-8 kits | DOJINDO | CK04 |
| BALB/c nude mice | Guangxi medical unniversity | Four weeks male mice |
| RIP™ RNA-Binding Protein Immunoprecipitation Kit | Millipore | 17-701 |
| RNA-Protein Pull-Down Kit | Thermo Fisher Scientific | 20164 |
| Oil Red O | Sorlabio | G1260 |
| bicinchoninic acid assay kit | Beyotime | P0010S |
| EpiQuik m6A RNA Methylation Quantification Kit | Epigentek, Colorimetric | P-9005-96 |
| Triglyceride assay kit | Biovision | K614-100 |
| Cholesterol assay kit | Biovision | K582-100 |
