# Supplementary material for: LINC01468 drives NAFLD-HCC progression through CUL4A-linked degradation of SHIP2
Source: Cell Death Discov. 2022 Nov 7;8:449. doi: 10.1038/s41420-022-01234-8 (PMC9640567; doi:10.1038/s41420-022-01234-8)

Unprocessed WB images for Figure 1

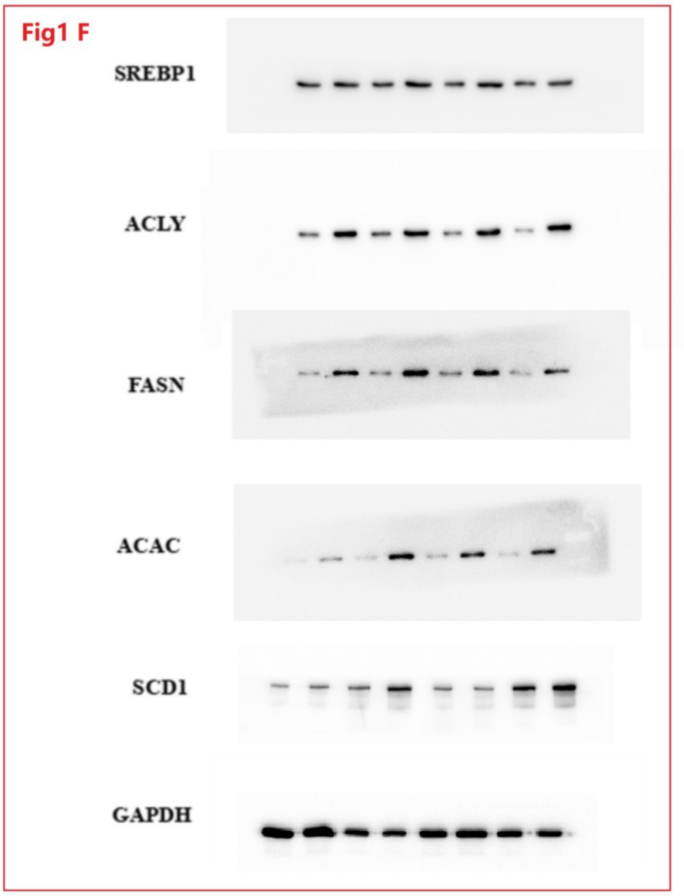

Unprocessed WB images for Figure 3

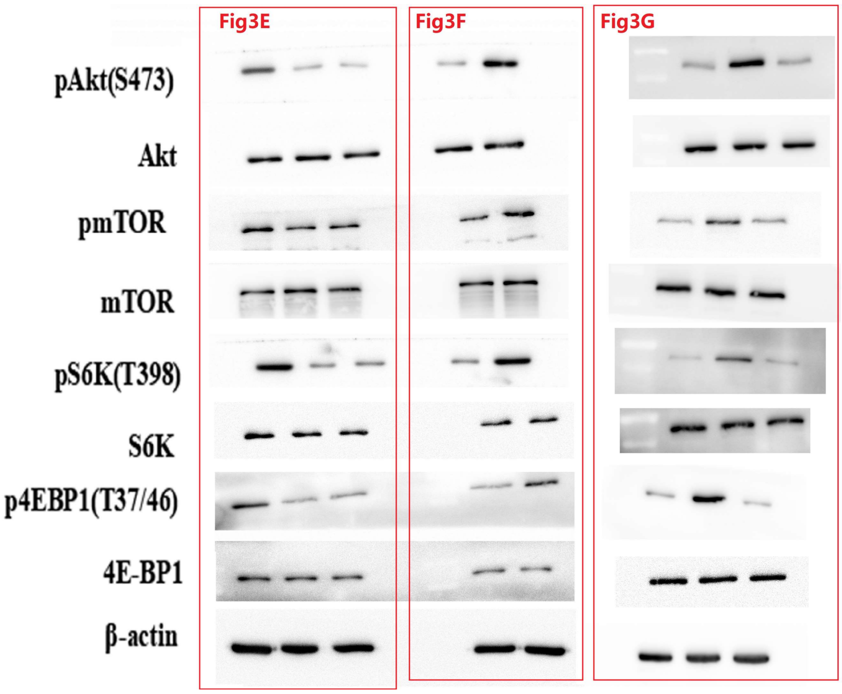

Unprocessed WB images for Figure 4

Fig 4C

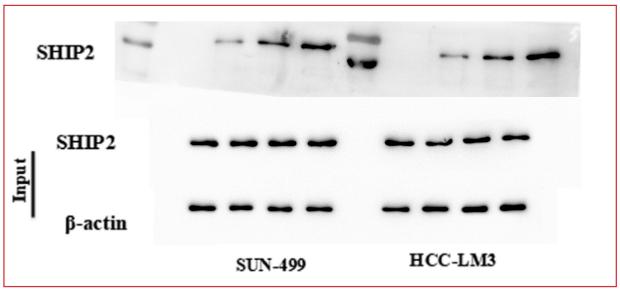

Fig4G

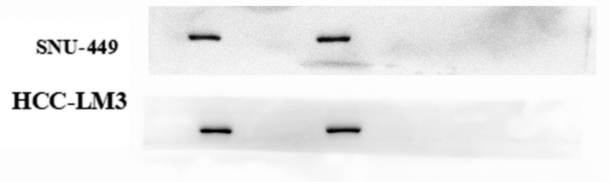

Fig4 I

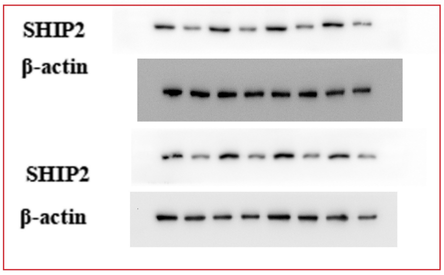

Fig 4K

Fig 4L

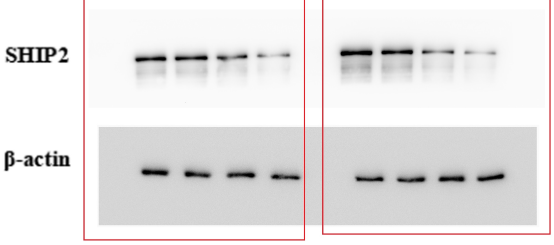

Unprocessed WB images for Figure 5

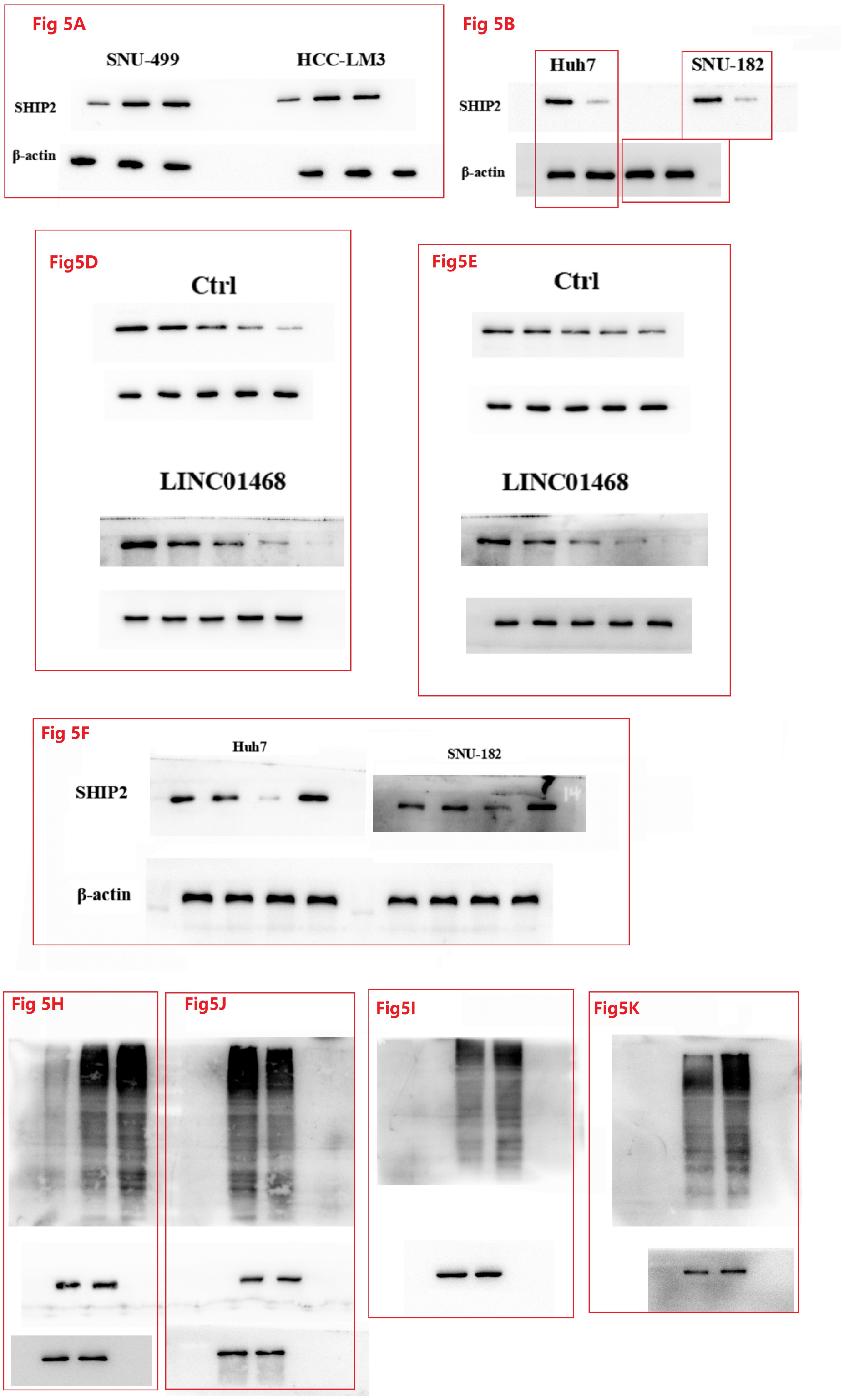

Unprocessed WB images for Figure 6

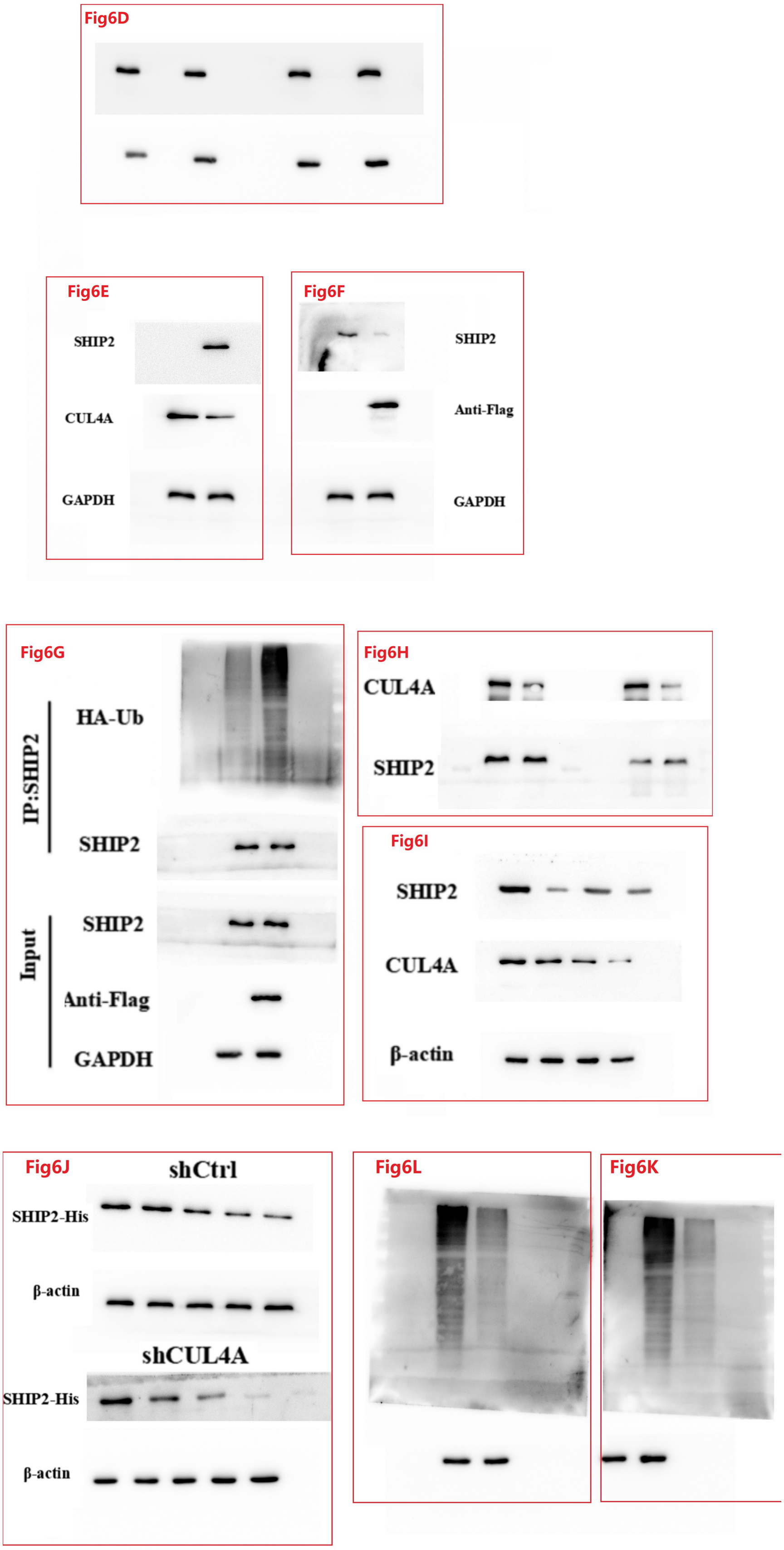

Unprocessed WB images for Figure 7

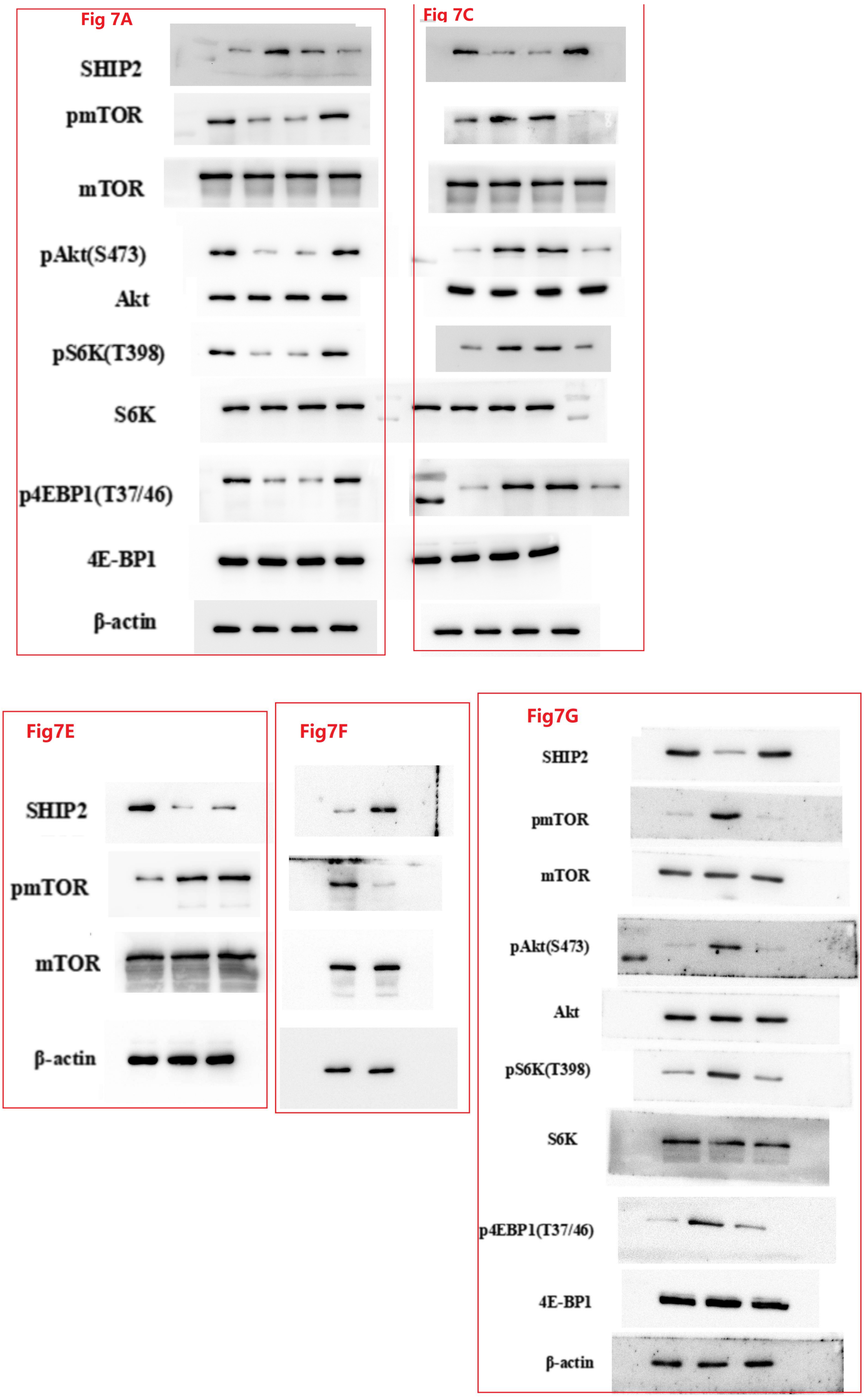

Fig1 F

Repeat 2

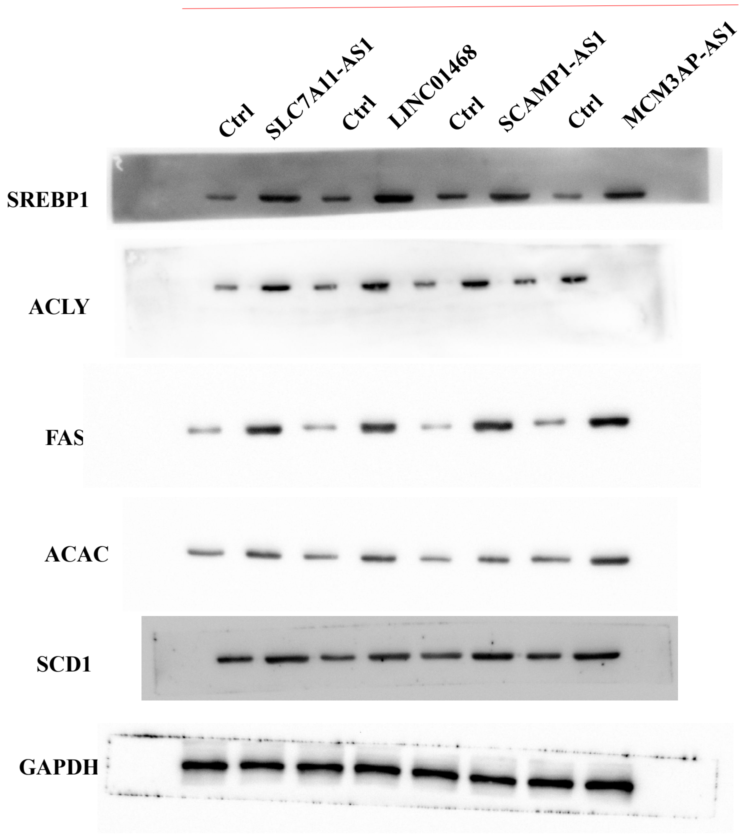

Fig1 F

Repeat 3

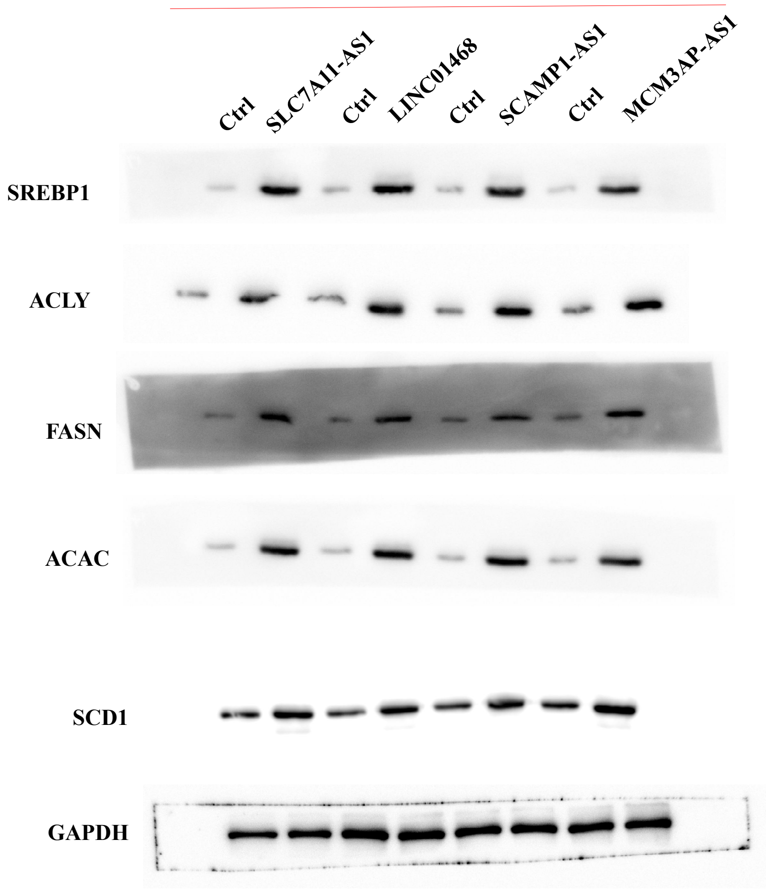

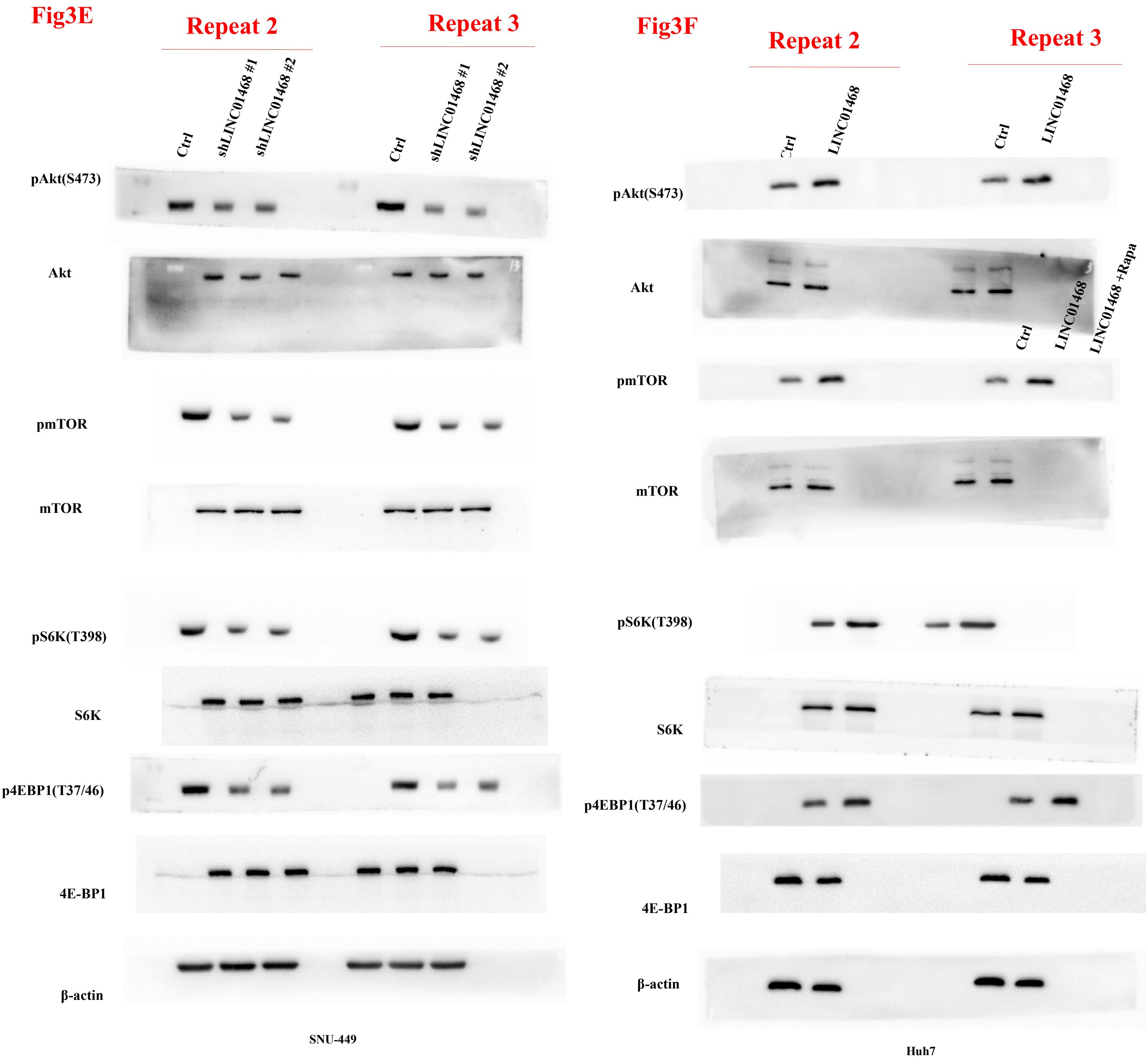

Fig3E
Repeat 2
Repeat 3
Ctrl
shLINC01468 #1
shLINC01468 #2
Ctrl
shLINC01468 #1
shLINC01468 #2
pAkt(S473)
Akt
pmTOR
mTOR
pS6K(T398)
S6K
p4EBP1(T37/46)
4E-BP1
β-actin
SNU-449
Fig3F
Repeat 2
Repeat 3
Ctrl
LINC01468
Ctrl
LINC01468
Ctrl
LINC01468
LINC01468 +Rapa
pAkt(S473)
Akt
pmTOR
mTOR
pS6K(T398)
S6K
p4EBP1(T37/46)
4E-BP1
β-actin
Huh7

Fig3G

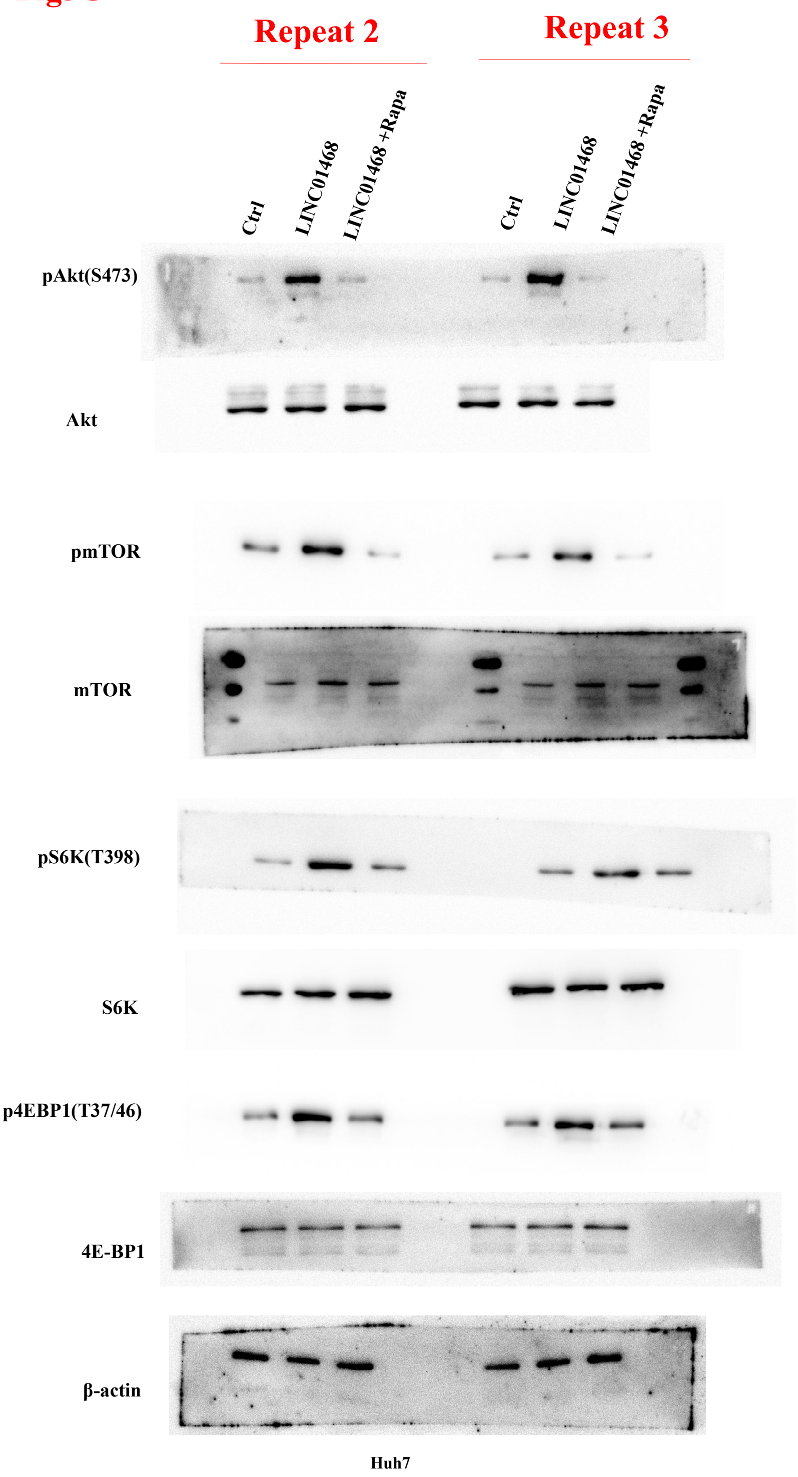

Repeat 2
Repeat 3
Ctrl
LINC01468
LINC01468 +Rapa
Ctrl
LINC01468
LINC01468 +Rapa
pAkt(S473)
Akt
pmTOR
mTOR
pS6K(T398)
S6K
p4EBP1(T37/46)
4E-BP1
β-actin
Huh7

Fig 4C

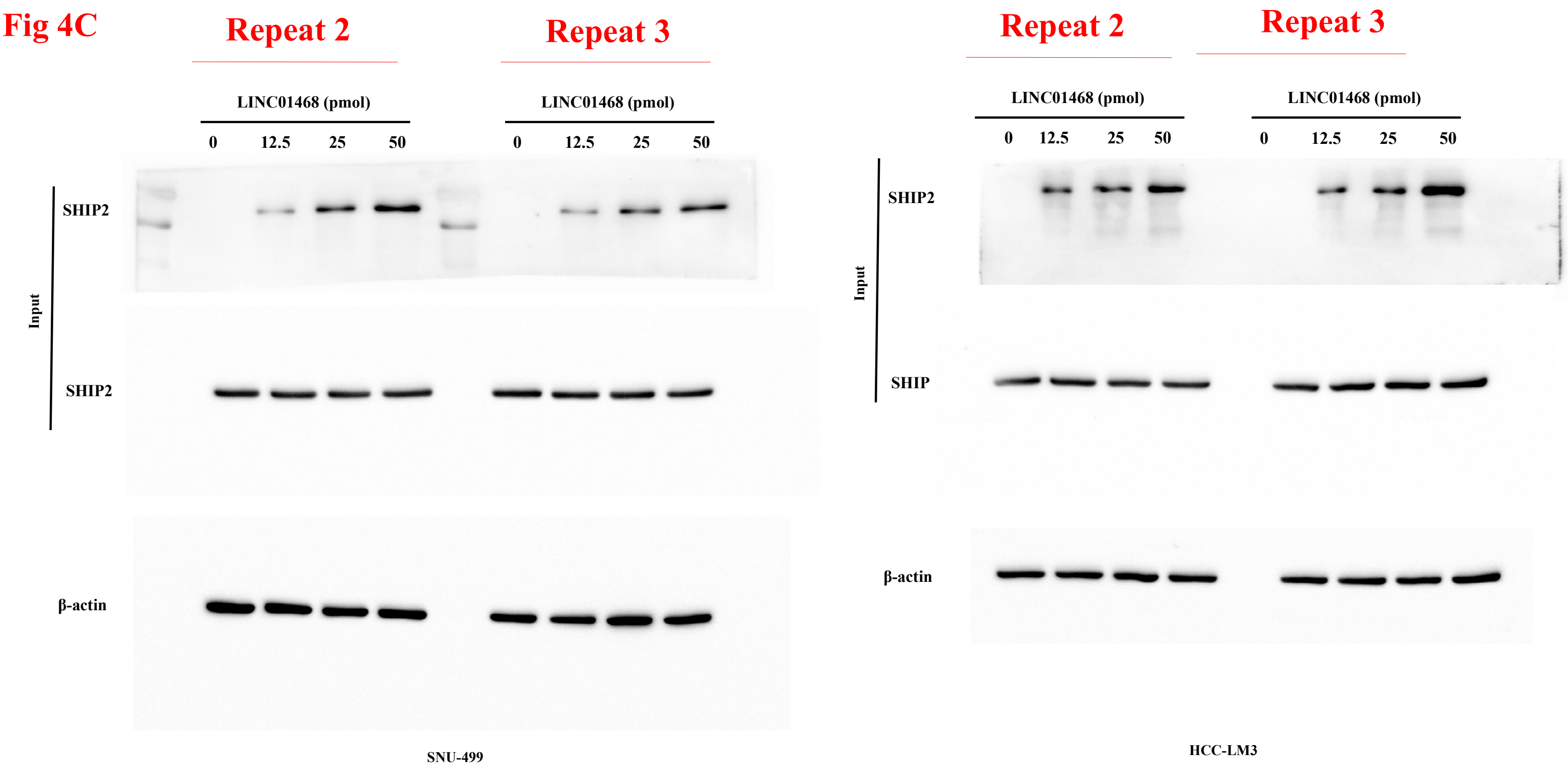
Repeat 2
Repeat 3
LINC01468 (pmol)
0
12.5
25
50
Input
SHIP2
SHIP2
β-actin
SNU-499
Repeat 2
Repeat 3
LINC01468 (pmol)
Input
SHIP2
SHIP
β-actin
HCC-LM3

Fig 4G

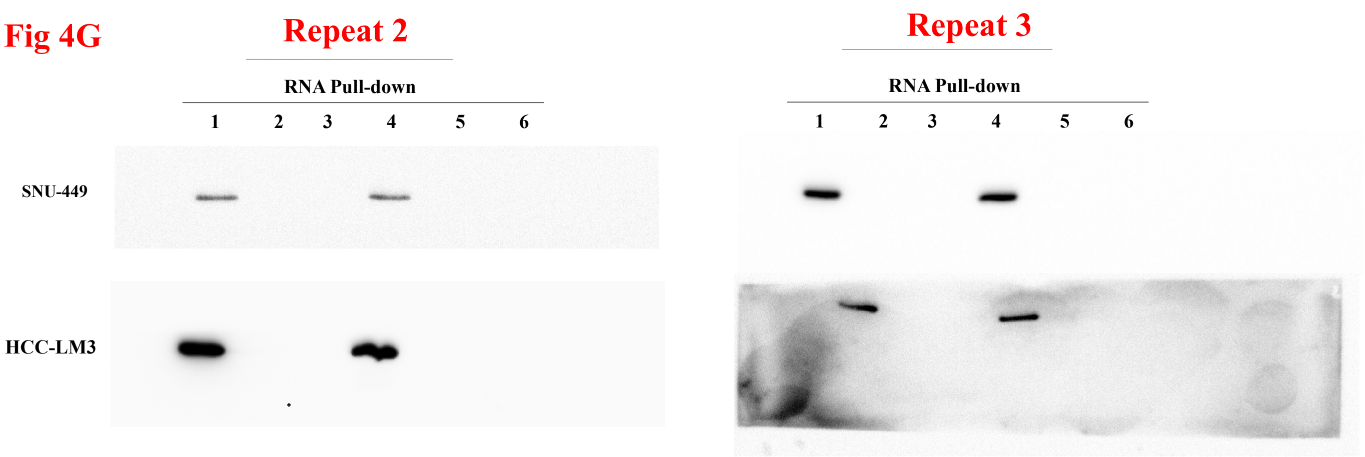
Repeat 2
RNA Pull-down
1 2 3 4 5 6
SNU-449
HCC-LM3
Repeat 3
RNA Pull-down
1 2 3 4 5 6

Fig 4I

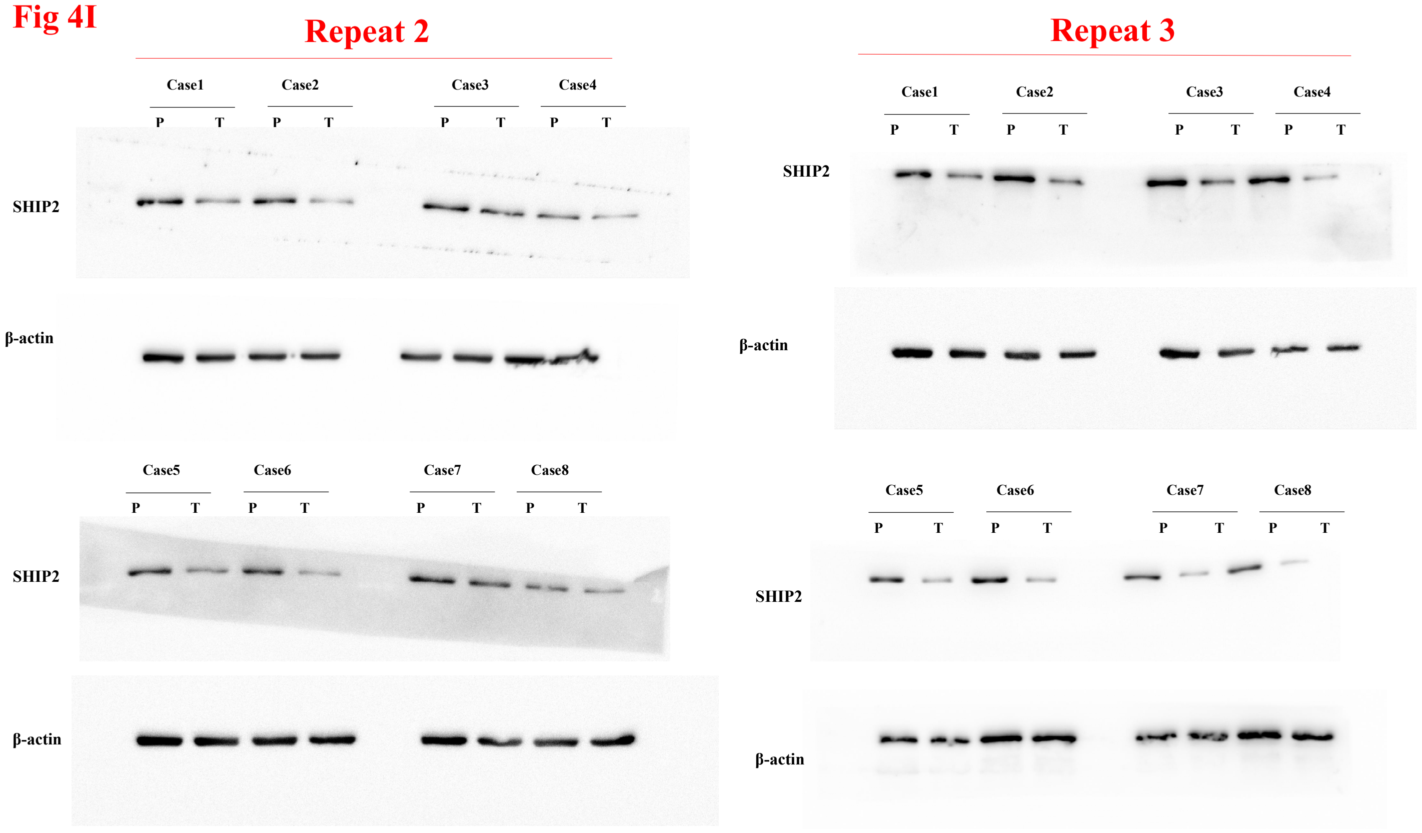

Fig 4K

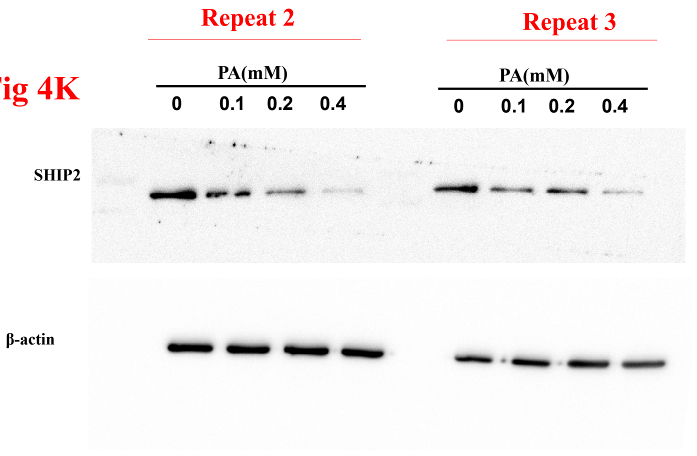

Fig 4L

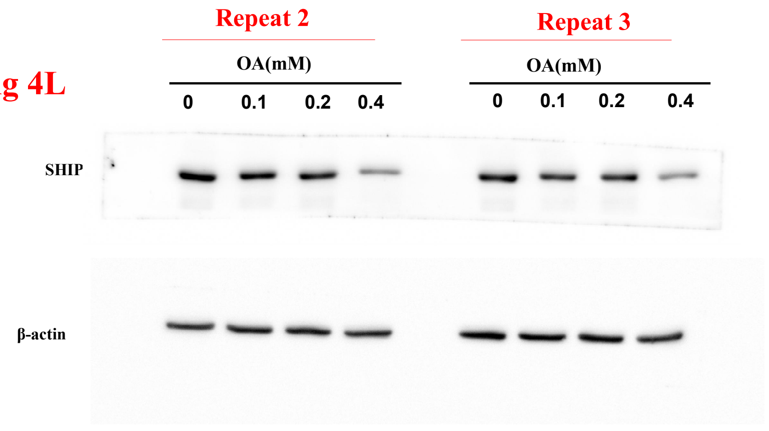

Fig 5A

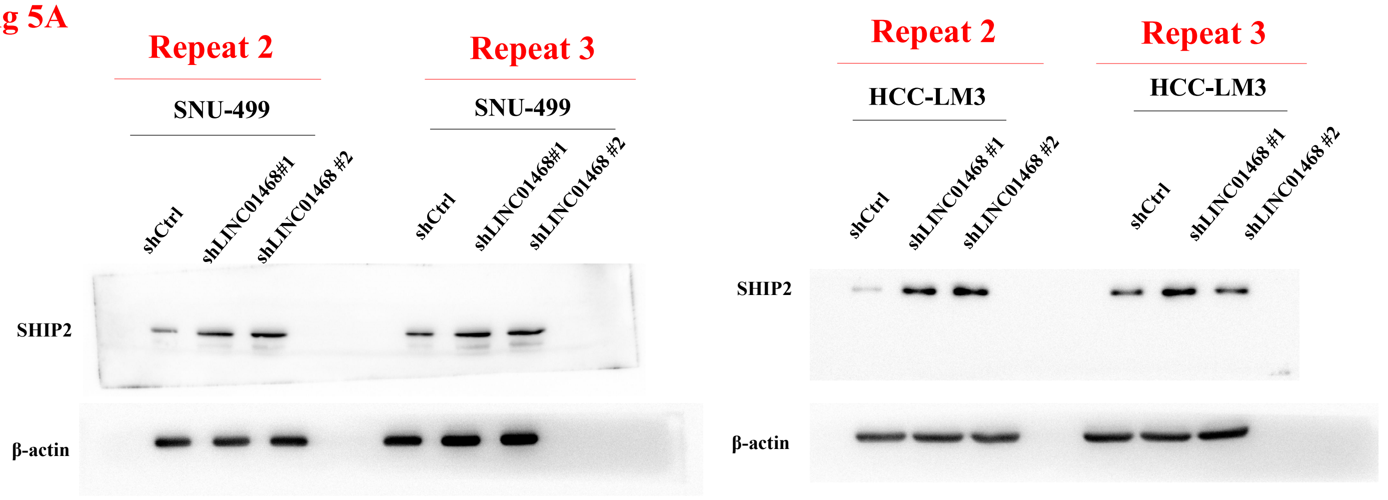

Fig 5B

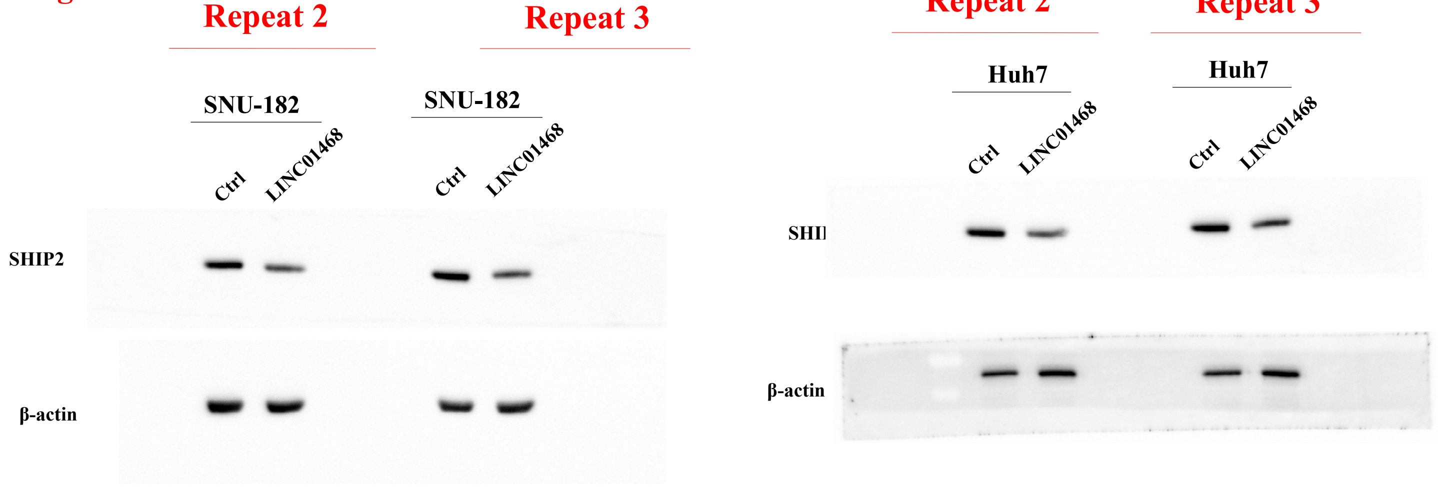

Fig 5D

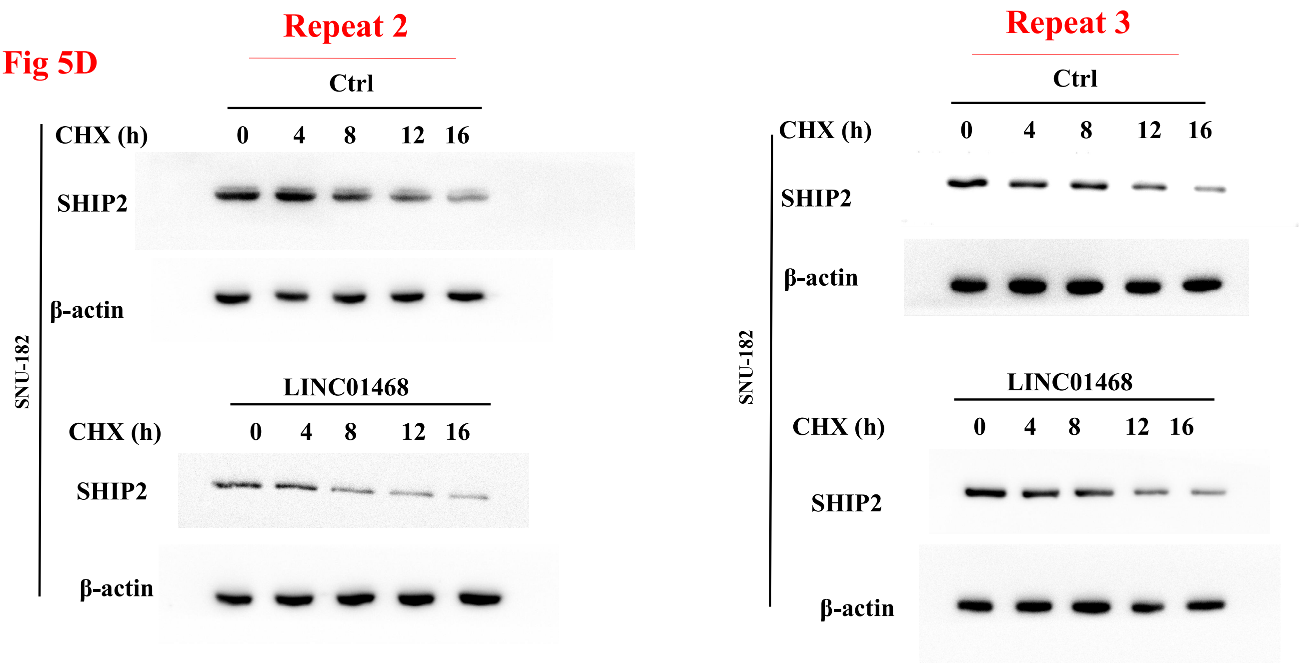

Fig 5E

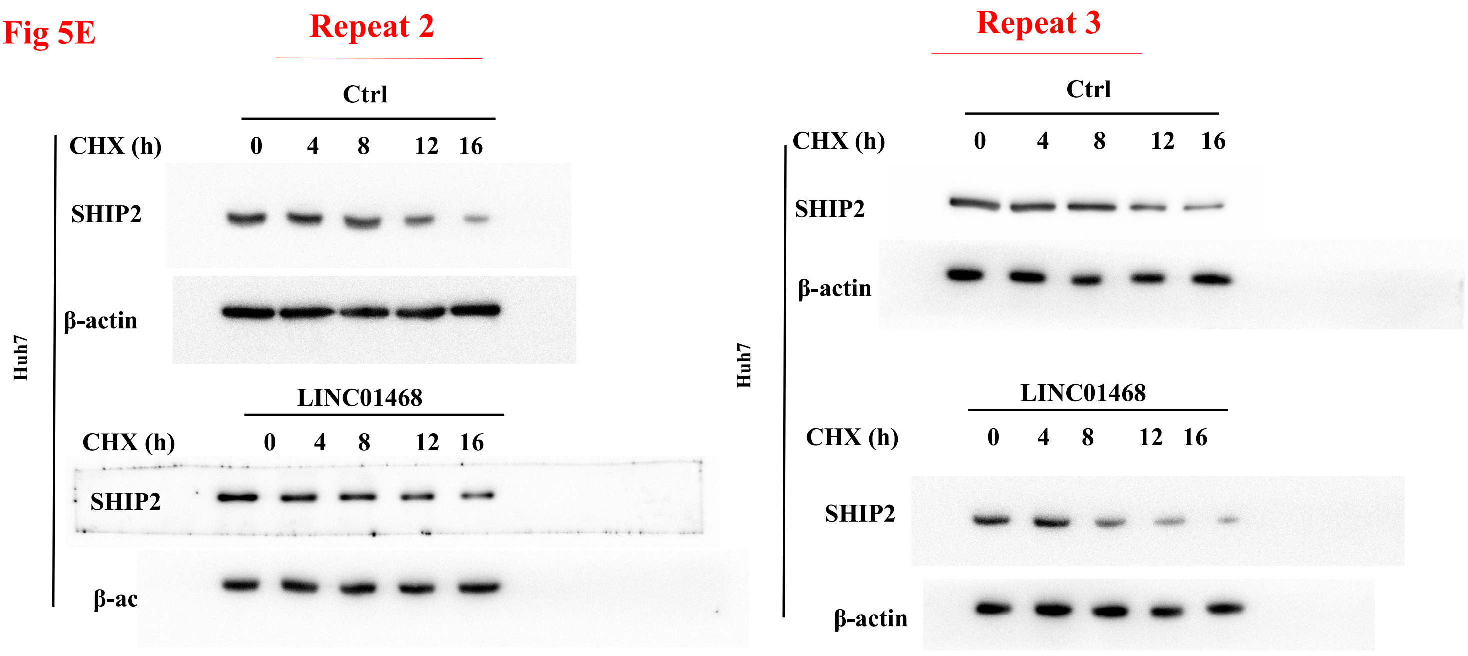
Repeat 2
Ctrl
CHX (h)
0 4 8 12 16
SHIP2
β-actin
Huh7
LINC01468
CHX (h)
0 4 8 12 16
SHIP2
β-ac
Repeat 3
Ctrl
CHX (h)
0 4 8 12 16
SHIP2
β-actin
Huh7
LINC01468
CHX (h)
0 4 8 12 16
SHIP2
β-actin

Fig 5F

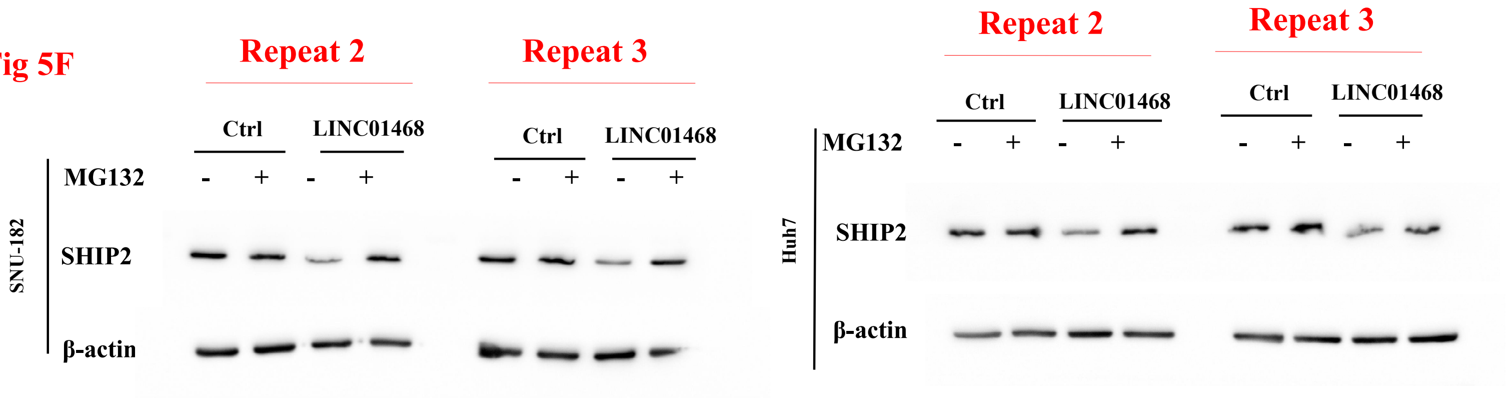
Repeat 2
Repeat 3
Ctrl
LINC01468
Ctrl
LINC01468
MG132
- + - +
- + - +
SNU-182
SHIP2
β-actin
Repeat 2
Repeat 3
Ctrl
LINC01468
Ctrl
LINC01468
MG132
- + - +
- + - +
Huh7
SHIP2
β-actin

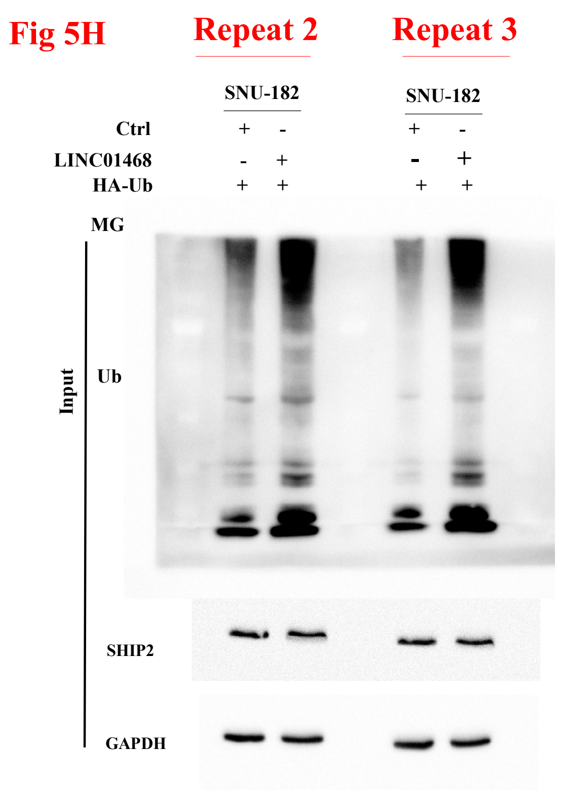
Fig 5H
Repeat 2
Repeat 3
SNU-182
SNU-182
Ctrl + - + -
LINC01468 - + - +
HA-Ub + + + +
MG
Input
Ub
SHIP2
GAPDH

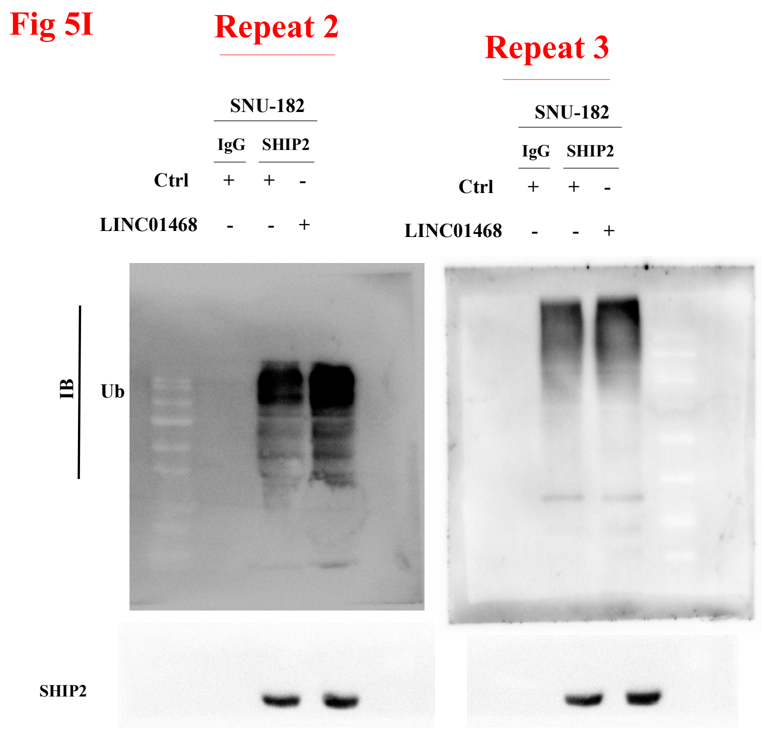
Fig 5I
Repeat 2
Repeat 3
SNU-182
IgG SHIP2
Ctrl + + -
LINC01468 - - +
SNU-182
IgG SHIP2
Ctrl + + -
LINC01468 - - +
IB
Ub
SHIP2

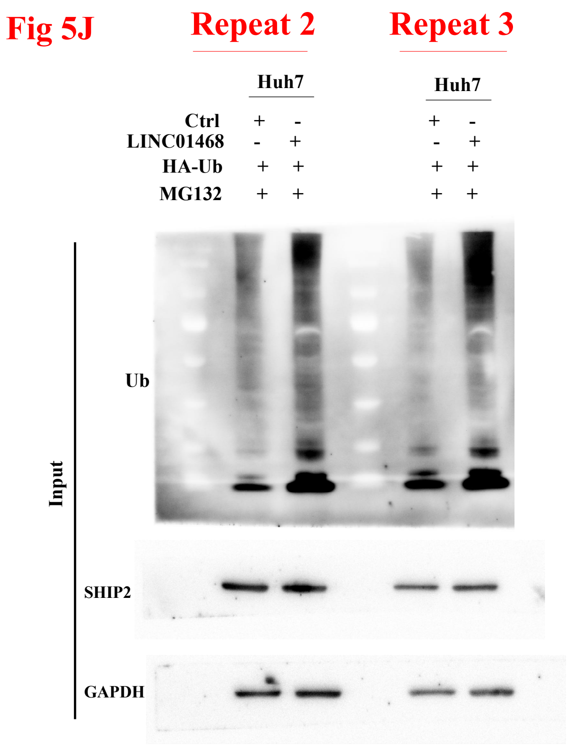
Fig 5J
Repeat 2
Repeat 3
Huh7
Huh7
Ctrl + - + -
LINC01468 - + - +
HA-Ub + + + +
MG132 + + + +
Input
Ub
SHIP2
GAPDH

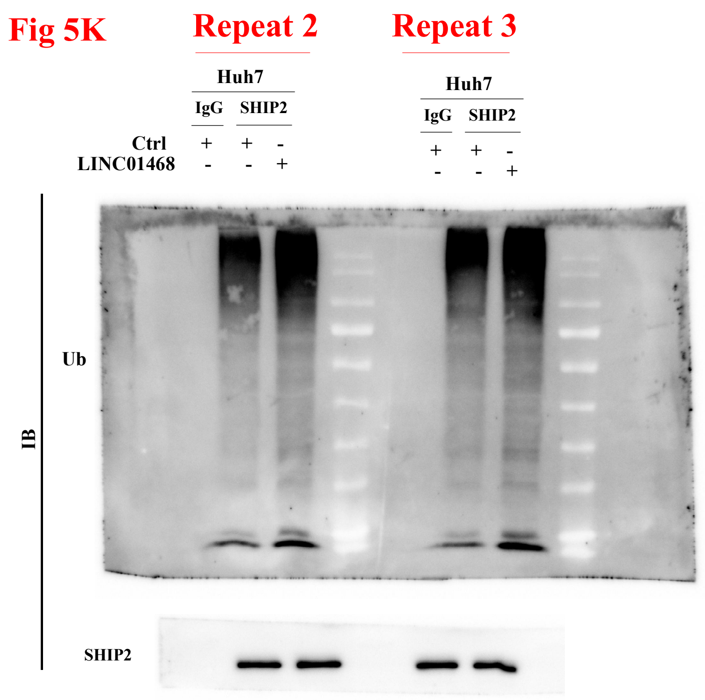
Fig 5K
Repeat 2
Repeat 3
Huh7
IgG SHIP2
Ctrl + + -
LINC01468 - - +
Huh7
IgG SHIP2
+ + -
- - +
IB
Ub
SHIP2

Fig 6D

Repeat 2 **Repeat 3**

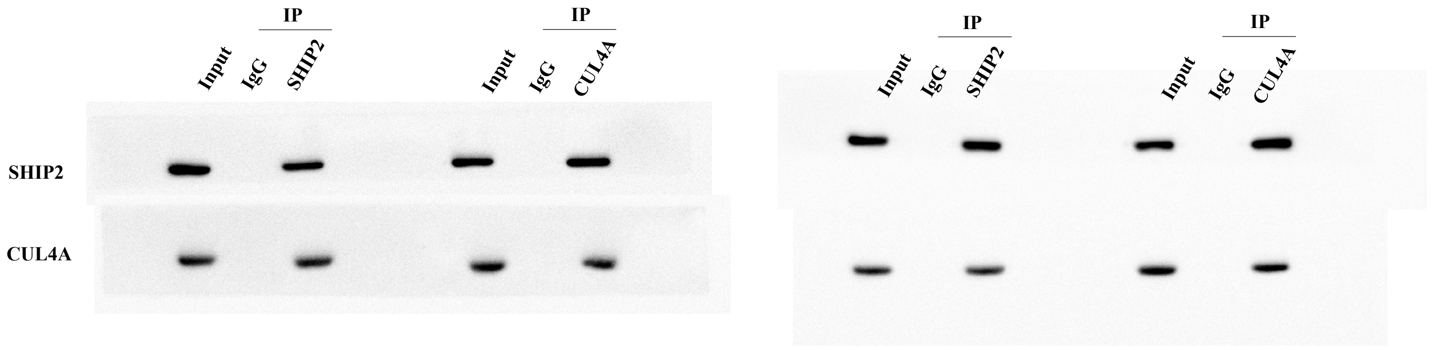

Fig 6E

Repeat 2 **Repeat 3**

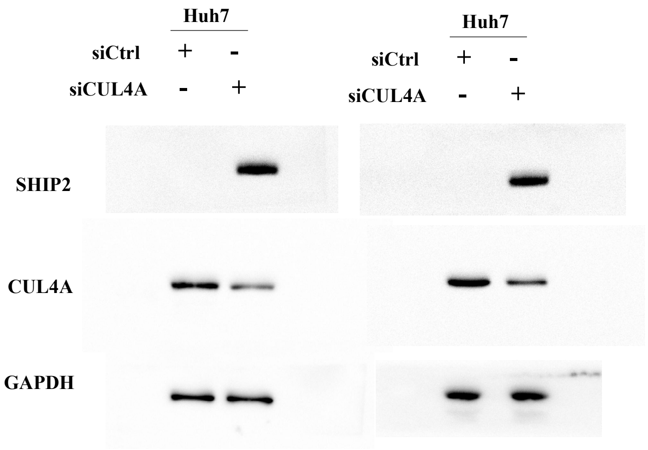

Fig 6F

Repeat 2 **Repeat 3**

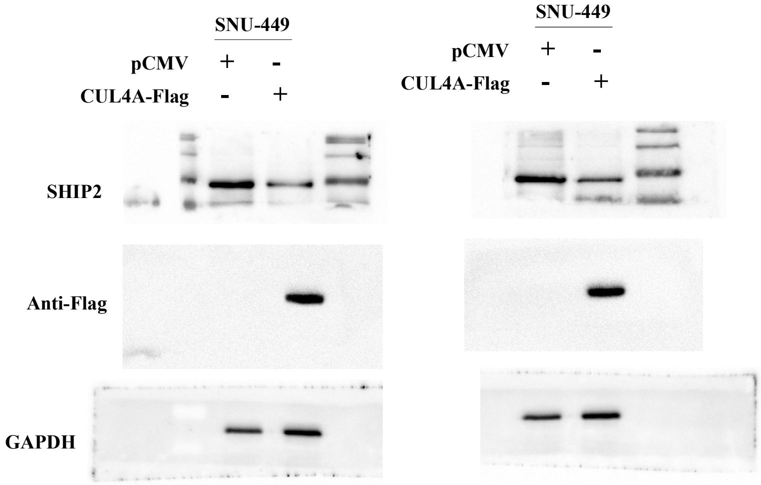

Fig 6G

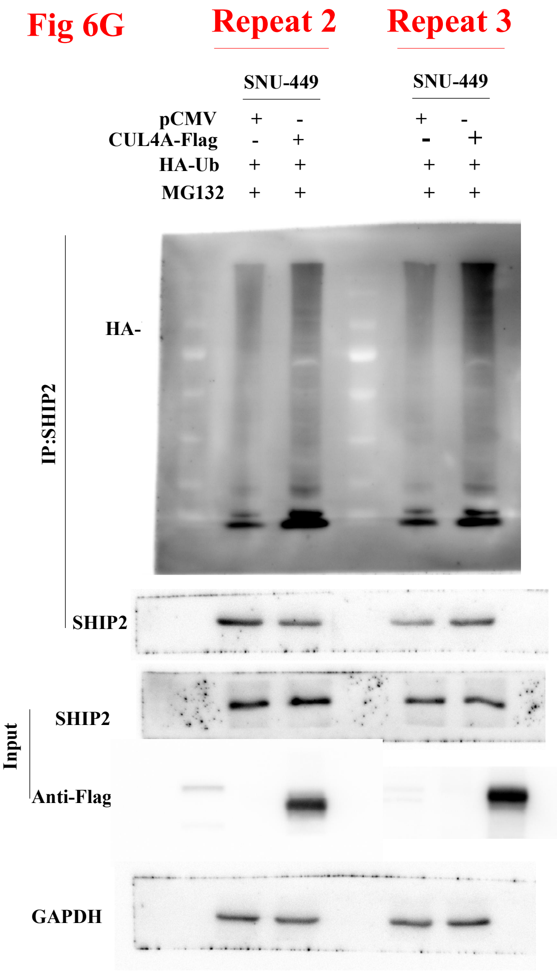

Fig 6H

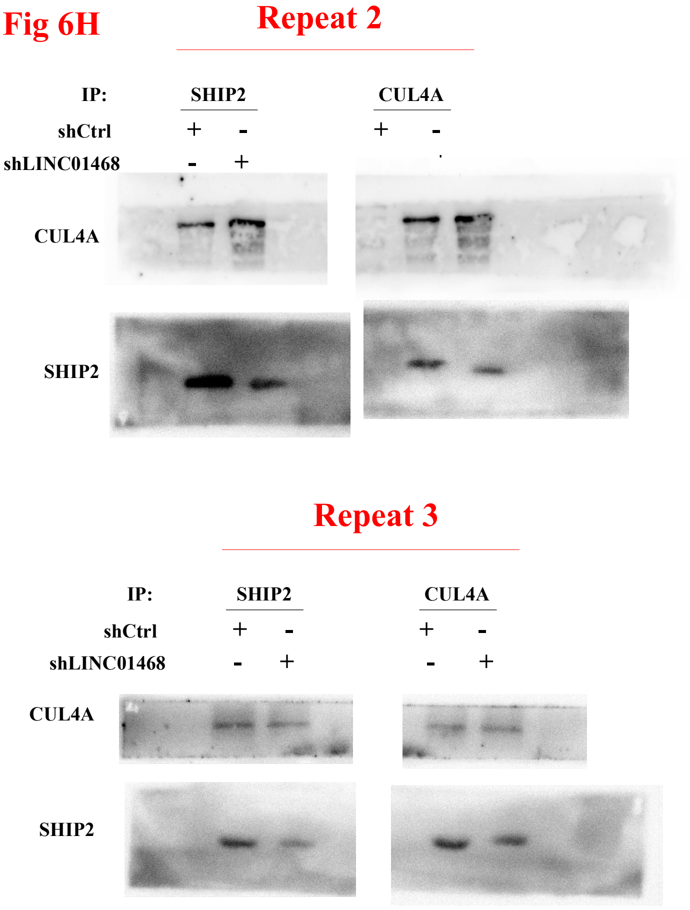

Fig 6I

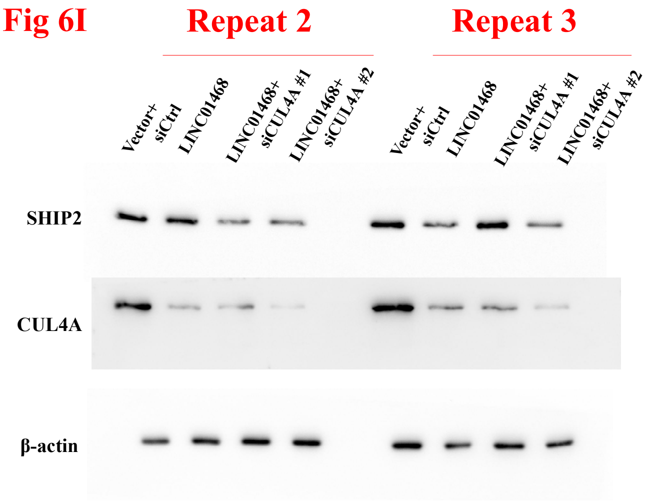

Fig 6J

Repeat 2 | Repeat 3

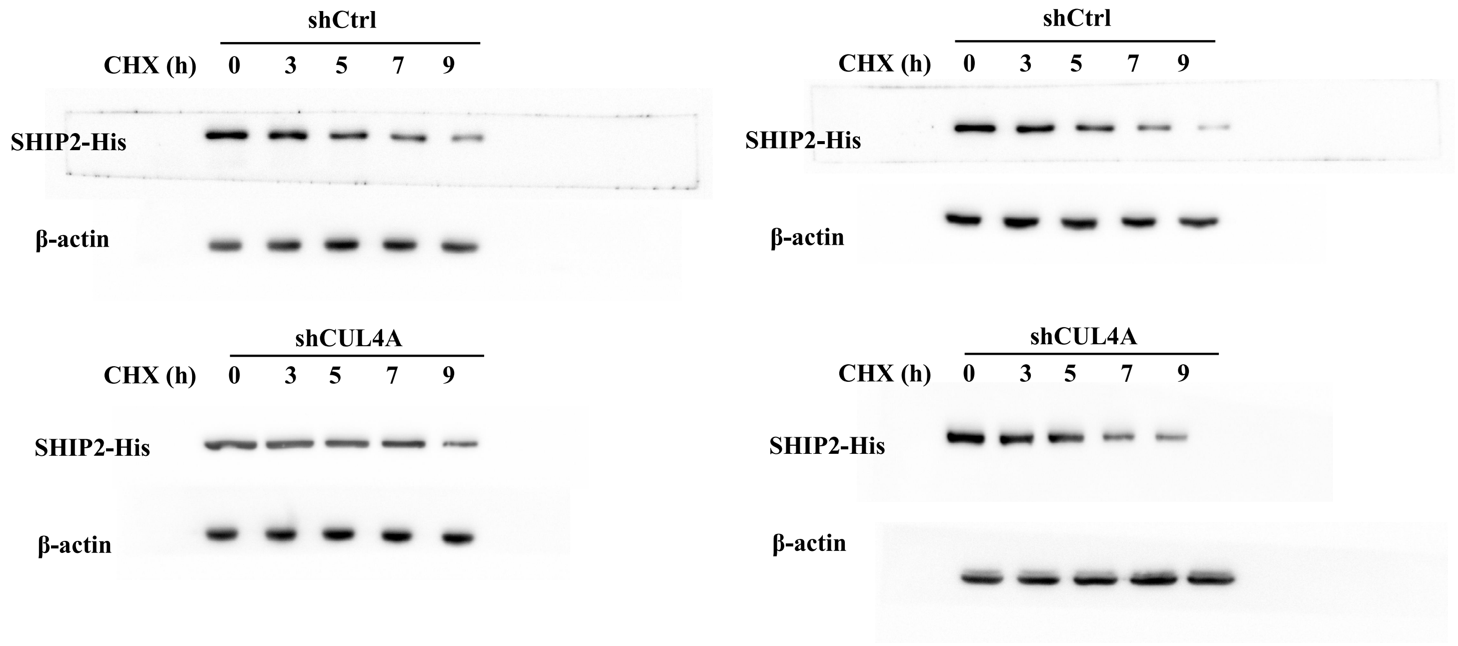

Fig6K

Repeat 2 | Repeat 3

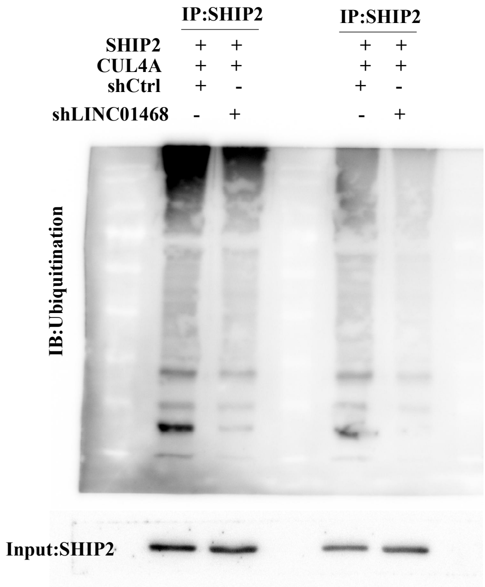

Fig6L

Repeat 2 | Repeat 3

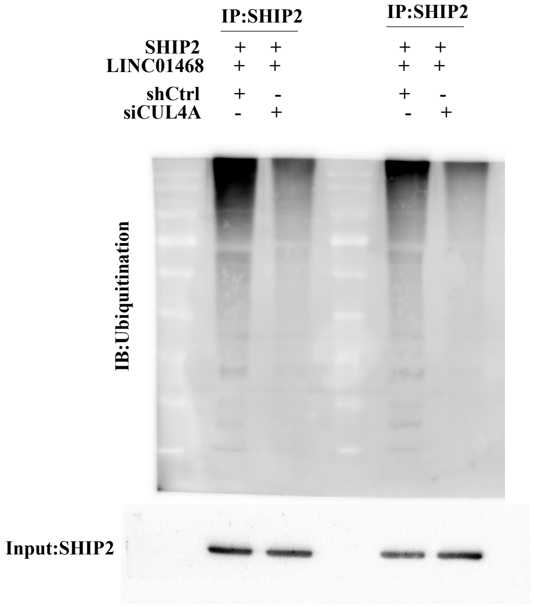

Fig7A

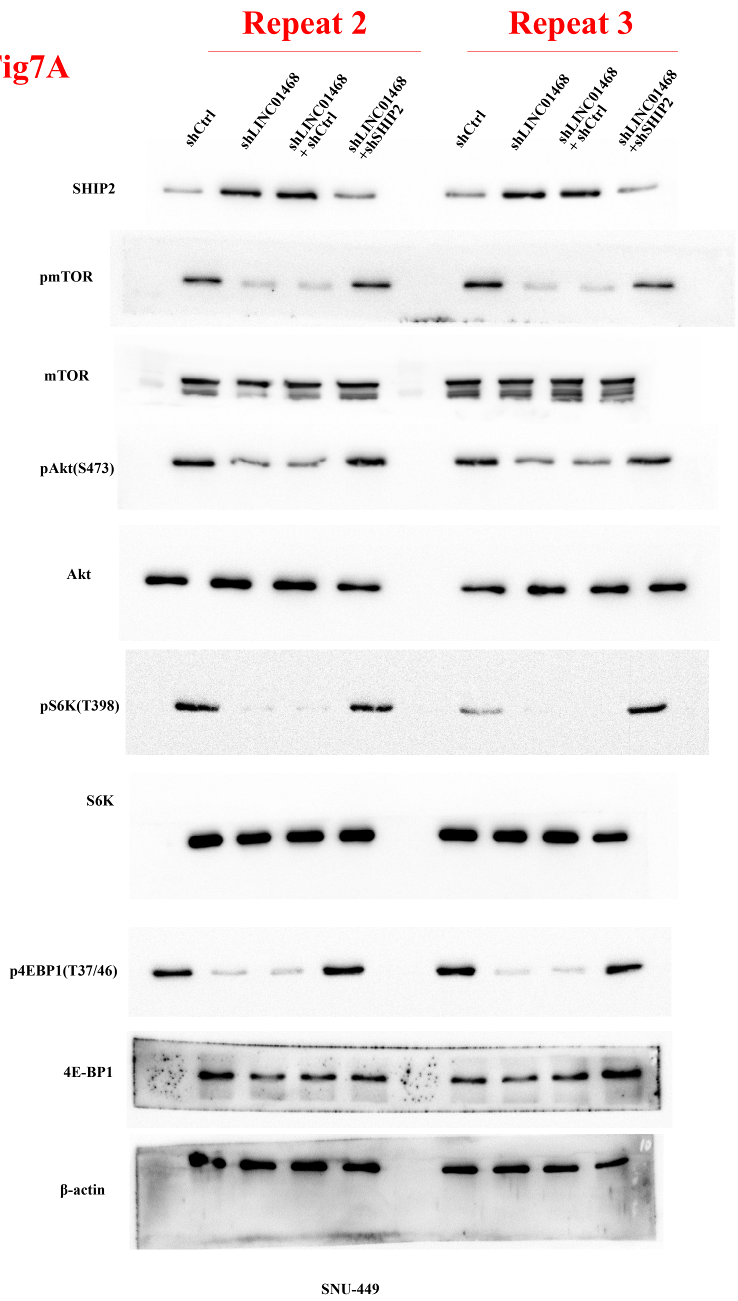

Repeat 2
Repeat 3
shCtrl
shLINC01468
shLINC01468 + shCtrl
shLINC01468 +shSHIP2
shCtrl
shLINC01468
shLINC01468 + shCtrl
shLINC01468 +shSHIP2
SHIP2
pmTOR
mTOR
pAkt(S473)
Akt
pS6K(T398)
S6K
p4EBP1(T37/46)
4E-BP1
β-actin
SNU-449

Fig7C

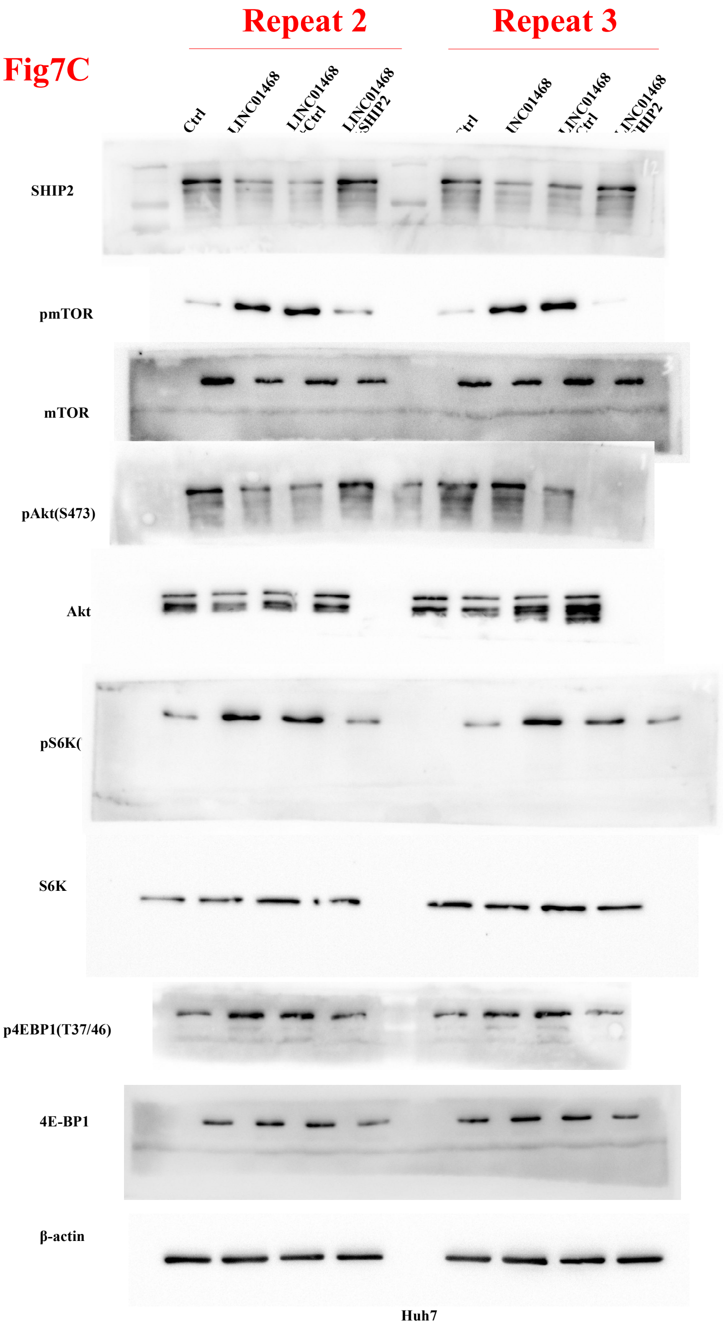

Repeat 2
Repeat 3
Ctrl
LINC01468
LINC01468 Ctrl
LINC01468 SHIP2
INC01468
SHIP2
pmTOR
mTOR
pAkt(S473)
Akt
pS6K(
S6K
p4EBP1(T37/46)
4E-BP1
β-actin
Huh7

Fig 7E

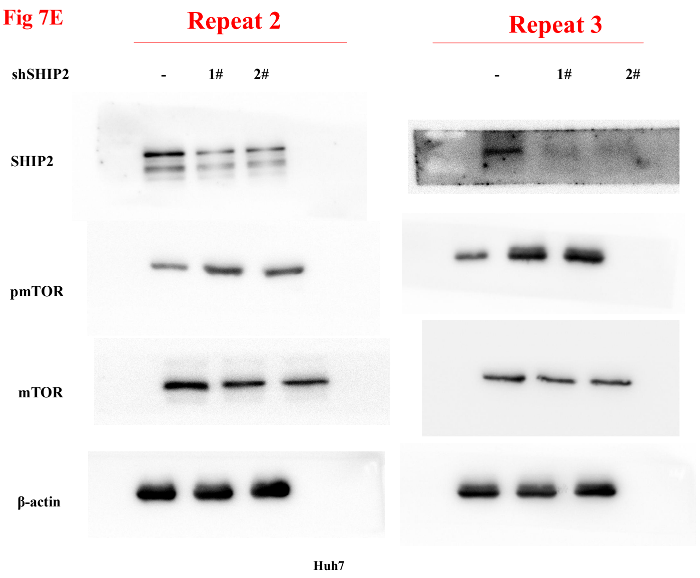

Fig7F

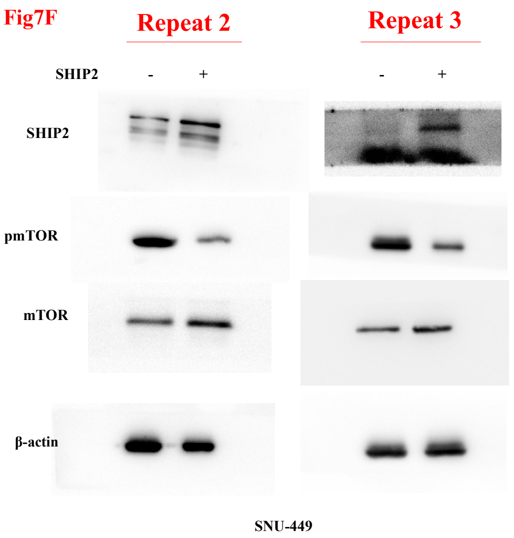

Fig 7G

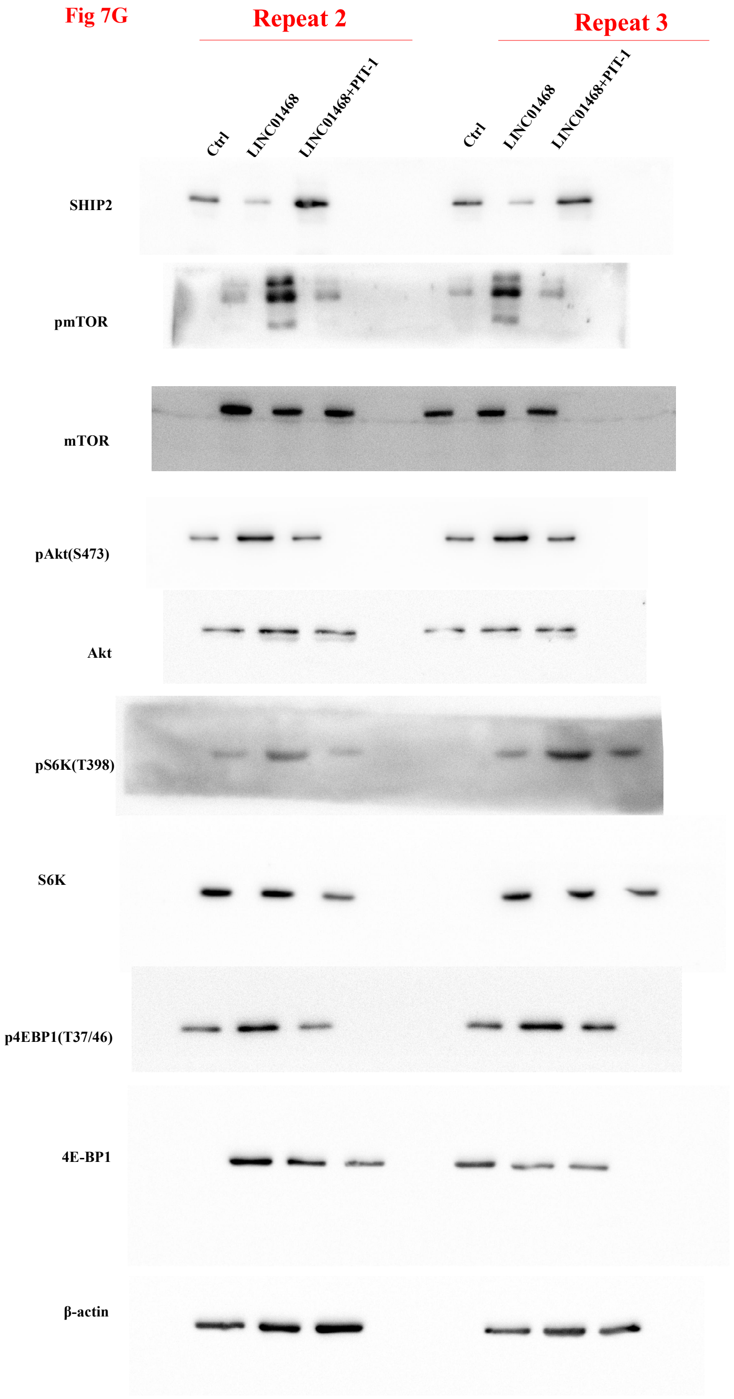

Supplement: Supplementary file 4 — Unprocessed WB [file 41420_2022_1234_MOESM4_ESM.pdf]
